# Environmental and molecular analysis of the floral transition in the lower eudicot *Aquilegia formosa*

**DOI:** 10.1186/2041-9139-2-4

**Published:** 2011-02-17

**Authors:** Evangeline S Ballerini, Elena M Kramer

**Affiliations:** 1 Dept. of Organismic and Evolutionary Biology, Harvard University, 16 Divinity Ave., Cambridge, MA, 02138, USA; 2 Dept. of Genetics, University of GA, Fred. C. Davison Life Sciences Complex, Athens, GA, 30602, USA

## Abstract

**Background:**

Flowering is a critical transition in plant development, the timing of which can have considerable fitness consequences. Until recently, research into the genetic control of flowering time and its associated developmental changes was focused on core eudicots (for example, Arabidopsis) or monocots (for example, *Oryza*). Here we examine the flowering response of *Aquilegia formosa*, a member of the eudicot order Ranunculales that is emerging as an important model for the investigation of plant ecology and evolution.

**Results:**

We have determined that *A. formosa *has a strong vernalization requirement but little or no photoperiod response, making it a day neutral (DN) plant. Consistent with this, the *Aquilegia *homolog of *FLOWERING LOCUS T *(*AqFT*) is expressed in both long and short days but surprisingly, the locus is expressed before the transition to flowering. *In situ *hybridizations with homologs of several Arabidopsis Floral Pathway Integrators (FPIs) do not suggest conserved functions relative to Arabidopsis, the potential exceptions being *AqLFY *and *AqAGL24.2*.

**Conclusions:**

In *Aquilegia*, vernalization is critical to flowering but this signal is not strictly required for the transcriptional activation of *AqFT*. The expression patterns of *AqLFY *and *AqAGL24.2 *suggest a hypothesis for the development of *Aquilegia*'s determinate inflorescence whereby their differential expression controls the progression of each meristem from inflorescence to floral identity. Interestingly, none of the *Aquilegia *expression patterns are consistent with a function in floral repression which, combined with the lack of a *FLC *homolog, means that new candidate genes must be identified for the control of vernalization response in *Aquilegia*.

## Background

The genus *Aquilegia *(Ranunculaceae, Ranunculales) is emerging as an important new model system for evolutionary, ecological, and developmental research [1-3]. *Aquilegia *(columbine) is a member of an early diverging lineage of the eudicots, arising prior to the genome duplication that occurred at the base of the core eudicot radiation [4,5]. The genus consists of approximately 70 species that have diversified across the northern hemisphere over the past 1 to 5 million years, exhibiting wide variation in floral morphology and ecological habitat [6-8]. The rapid speciation of the genus is linked to shifts in pollinator syndromes, which include changes in flower color, orientation and, perhaps most importantly, nectar spur morphology [9,10]. Several characteristics make *Aquilegia *amenable to genetic research. Plants are self-compatible and their recent, rapid speciation has left most species in the genus capable of inter-breeding, providing a useful tool for carrying out QTL analyses [11]. In addition, *Aquilegia *has a relatively small diploid genome (n = 7, approximately 300 Mbp; [3], S. A. Hodges, pers. comm.) for which several genomic tools, such as two fingerprinted Bacterial Artificial Chromosome (BAC) libraries and an Expressed Sequece Tag (EST) database, have already been developed. A complete genome sequence has recently been released http://www.phytozome.net/ and the compilation of detailed genetic and physical maps is currently underway. In addition, virus induced gene silencing (VIGS) has been successfully utilized in *Aquilegia *for transient gene knockdown [12,13]. One drawback of many *Aquilegia *species, however, is their relatively long generation time, which is tied in part to a slow transition to flowering.

The transition from vegetative to reproductive growth marks a key developmental stage in a plant's life history. Flowering time has a direct connection to reproductive fitness and variation in the process can lead to reproductive isolation, an important factor in the speciation process [14-17]. Factors affecting flowering time are, therefore, likely to be strong components influencing adaptation of plant populations to their environments. While many factors can affect flowering time, including water and nutrient availability, stress, and hormone signaling, two of the primary environmental factors influencing flowering time - photoperiod and temperature - are highly associated with seasonality. Flowering time in plants is most often categorized based on responses to these seasonal cues. Regarding photoperiod, plants are categorized as long day (LD), short day (SD), or day neutral (DN) depending on which photoperiodic conditions promote flowering. One of the most common temperature responses is that of vernalization, whereby plants flower in response to an extended exposure to cold temperatures. In nature, such a requirement ensures that a plant does not flower until after winter, which is particularly relevant for a temperate genus such as *Aquilegia*.

**Figure 1:**
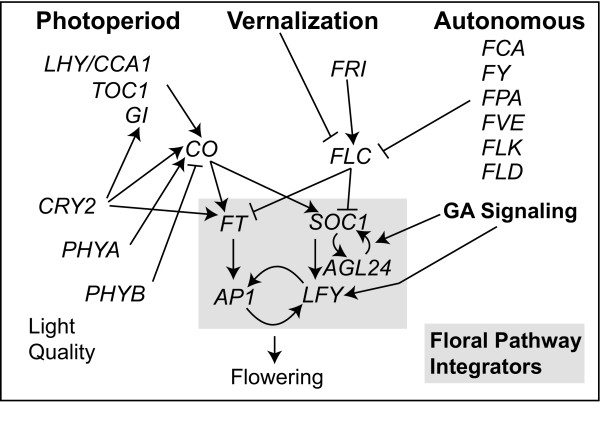
**A simplified schematic showing flowering time gene interactions in *Arabidopsis thaliana***. Based on [28,32,37,89-91]. Floral Pathway Integrators are highlighted in grey.

To date, the genetics of flowering time control have been most extensively studied in Arabidopsis (*Arabidopsis thaliana*). In Arabidopsis, flowering occurs when a group of genes known as the Floral Pathway Integrators (FPI) are up-regulated in the shoot apical meristem (SAM). These genes integrate signals from the photoperiod, vernalization, autonomous, and GA pathways to induce the expression of floral meristem identity genes and produce a flower. Figure 1 provides a simplified schematic overview of this genetic network, showing the rather complex interactions that exist between the FPI genes. Following environmental induction of flowering, a cascade of gene expression is induced in the plant apex to confer inflorescence and floral meristem identity [18]. In the apical meristem, *FLOWERING LOCUS T *(*FT*), a mobile floral signal produced in leaves, induces expression of the genes *SUPPRESSOR OF OVEREXPRESSION OF CONSTANS 1 (SOC1) *and *AGAMOUS*-*LIKE 24 (AGL24)*, which confer inflorescence meristem identity on the apex [19,20]. *SOC1 *and *AGL24 *physically interact and positively regulate both each other and *LEAFY *(*LFY) *in the lateral meristems of the inflorescence [21,22]. *LFY *contributes to the induction of *APETALA1 *(*AP1*), which itself positively feeds back on *LFY *[23,24]. Together, *LFY *and *AP1 *confer floral meristem identity (FMI) on the lateral meristems, with *LFY *interacting with various cofactors to regulate the expression of floral homeotic genes in specific regions of the floral meristem and *AP1 *acting to suppress continued *AGL24 *expression in the FM [25-27].

As a facultative LD plant, Arabidopsis flowers rapidly under LD conditions and many loci important to this response have been identified. Not surprisingly, several of these encode proteins involved in light perception, such as the phytochromes (*PHYA*, *PHYB*, *PHYC*, *PHYD*, *PHYE*) and the cryptochromes (*CRY1 *and *CRY2*), or genes that are critical to the maintenance of the central oscillator of the circadian clock (*LHY*, *CCA1*, *TOC1*, *GI*) (reviewed in [28]). Signals perceived by the photoreceptors and genetic input from the central oscillator are integrated to allow plants to sense changes in daylength. The gene *CONSTANS *(CO) is important for this integration process and directly regulates *FT *transcription in a positive manner to induce flowering under inductive long days [29]. The expression of *CO *mRNA is positively regulated by *GIGANTEA *(*GI*), which is a central member of the clock and has a circadian expression pattern [30,31]. Work by Valverde *et al*. [32] highlighted the important roles of the photoreceptors in CO protein stability under different light regimes, demonstrating that CO protein is degraded in darkness. Thus, in Arabidopsis, the coincidence of *CO *mRNA expression, CO protein stability, and resultant *FT *up-regulation only occurs during LD conditions, making Arabidopsis a facultative LD plant.

In addition to inductive long days, many Arabidopsis ecotypes exhibit a vernalization requirement. Naturally occurring mutations in the vernalization pathway result in the rapid cycling lines that are often used for genetic research [33-35]. While the photoperiod pathway is responsible for promoting flowering, the vernalization pathway is inhibitory and in Arabidopsis, this inhibition is largely mediated by the MADS-box gene *FLOWERING LOCUS C *(*FLC*) [36]. FLC has been shown to bind the promoter regions of the FPI genes *FT *and *SOC1*, presumably preventing their up-regulation by *CO *[36]. Before vernalization, *FLC *expression levels are high while those of *SOC1 *and *FT *are low [36]. These high levels of *FLC *are largely due to the activity of *FRIGIDA *(*FRI*), which has recently been shown to promote positive chromatin changes at the *FLC *locus [36]. Natural variation at the *FRI *locus produces ecotypes showing reduced *FLC *expression and rapid flowering without vernalization [33]. In ecotypes with functional alleles of *FRI *and *FLC*, several Polycomb Repressive Complex 2 components as well as associated proteins, including VERNALIZATION INSENSITIVE 3 (VIN3), VERNALIZATION 1 (VRN1), and VERNALIZATION 2 (VRN2), act during vernalization to alter the structure of the *FLC *locus, leaving it in a stably repressed state [36]. Upon returning to inductive long day conditions after vernalization, promotional signals from the photoperiod pathway can then induce expression of *FT*, *SOC1 *and the other FPIs. In the event that a plant does not experience environmental inductive signals to flower, the autonomous pathway helps ensure that it still has the opportunity to reproduce. In Arabidopsis, members of the autonomous pathway, including *FCA*, *FY*, *FPA*, *FVE*, and *FLOWERING LOCUS K (FLK)*, reduce *FLC *RNA levels via RNA metabolism or chromatin-state regulation [37,38].

While studies in Arabidopsis have provided valuable insight into the genetic regulation of flowering time (especially regarding flowering in response to environmental stimuli), there is tremendous variation in the flowering time trait across flowering plants. Studying the trait in diverse taxa will establish a comparative framework from which we can gain a better understanding of how the genetics of flowering time evolved. To date, genetic information on the regulation of flowering is largely limited to other core eudicot taxa such as pea, tomato, poplar, cucurbit, and morning glory, and to several of the monocot cereals such as rice, wheat, and barley. As a basal eudicot, studying flowering time in the genus *Aquilegia *will add an intermediate point of comparison between the core eudicots and the monocots.

Flowering time in the genus *Aquilegia *has been examined to a limited extent by researchers working to improve horticultural practices. Data from these papers are often conflicting and are confounded by the use of hybrid varieties that may exhibit atypical phenotypes. One constant, however, is that *Aquilegia *varieties show a strong response to vernalization after reaching floral competency around the 12 to 15-leaf stage [39]. This requirement is also observed in the main focus of recent genetic research, the species *Aquilegia formosa *(S.A. Hodges pers. comm.). Distributed throughout western North America, *Aquilegia formosa *has both broad latitudinal and altitudinal ranges, extending from Baja California to Alaska and from sea level to approximately 3,000 feet in elevation. This distribution includes populations that experience varied photoperiod and temperature regimes. Considerable genetic and genomic research has recently focused on populations of *A. formosa *found at approximately 38° north latitude and approximately 2,500 meters above sea level [3,40]. These high altitude populations are buried under snow from November through May or June, depending on winter precipitation, and typically flower in July through August. Despite these field observations, no controlled experiments have ever been conducted on flowering time response in *A. formosa *to determine what environmental signals control this transition. Since many genetic aspects of flowering time response appear to be broadly conserved, an important first step in studying the process is to identify *Aquilegia *homologs of the Arabidopsis loci and begin to study their regulation. The current study tackles both of these goals and finds that while photoperiod has a slight effect on flowering time in *A. formosa*, vernalization is the primary ecological factor promoting flowering in these populations. Analyses of the *Aquilegia *homologs of the Arabidopsis flowering time loci reveal some evidence of conservation, but a surprising degree of divergence. They have also provided insight into the developmental changes that define flowering in *Aquilegia*. This work now lays the foundation for all future studies of natural variation in *Aquilegia *flowering time response as well as providing a framework for manipulation of this process.

## Results

### Photoperiod and vernalization response in *Aquilegia formosa*

Based on the known phenology of *A. formosa*, we hypothesized that it would likely flower in response to long days and vernalization, similar to Arabidopsis. In order to test these hypotheses, we compared the flowering times of plants grown in controlled laboratory conditions under various photoperiod and vernalization regimes. Initial studies indicated a strong requirement for vernalization so we focused on comparing flowering time of plants vernalized and grown in short days (SD: 8L/16D) to that of plants vernalized and grown in long days (LD: 16L/8D).

Although *Aquilegia *is self-compatible, the use of isogenic lines is complicated by a strong susceptibility to inbreeding depression (S.A. Hodges, pers. comm.; [41]). Therefore, we used F2 hybrids of *Aquilegia formosa *collected from two populations located in eastern California and Nevada at roughly 2,500 meters above sea level. Plants were germinated and grown in either SD or LD conditions at 20°C until reaching approximately the 12 to 15 leaf stage at which point they were vernalized for eight weeks at 4°C in SD and then returned to their original SD or LD conditions.

**Figure 2:**
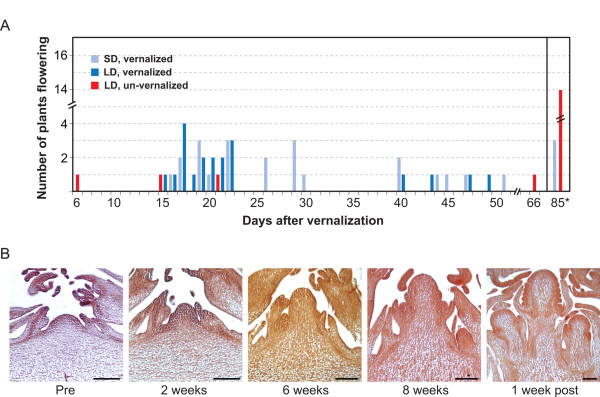
**Flowering time of *A. formosa *in different light regimes and meristematic development during vernalization**. **(A) **Flowering time of *A. formosa *plants grown under three different light/temperature regimes: constant SD with eight weeks of 4°C, LD with eight weeks of 4°C SD, or LD with eight weeks of 20°C SD. Day 1 is the first day that vernalized plants were removed from vernalization and LD non-vernalized plants were moved back into LD. Time to flowering was measured as the number of days following removal from vernalization when an inflorescence was visible above the leaf crown. The x-axis after 85 days when the experiment was ended. **(B) ***A. formosa *meristem development through vernalization. The SAM just prior to vernalization (Pre), two weeks into vernalization (two weeks), six weeks (six weeks) into vernalization, eight weeks into vernalization (eight weeks), and one week following vernalization (one week post).

We note that LD grown plants differed in appearance from their SD-grown siblings in that the latter showed much less leaflet and petiole expansion (See Additional File 1: Figure 1). On average, plants grown in SD took slightly longer to flower than plants grown in LD with mean days to flowering of 28.6 and 24.1 respectively, not including three SD plants that did not flower (Figure 2A). When the three non-flowering SD plants are included in a rank test analysis, the median flowering time three plants grown in SD and LD is 29 and 20 days, respectively, and the distribution of flowering time differs significantly (Mann-Whitney U = 2.3, n_1 _= 25, n_2 _= 20, P < 0.5 two-tailed). Dropping the three plants that did not flower, the median flowering time of SD plants drops to 26 days and the distributions of flowering time between SD and LD grown plants no longer differ significantly (Mann-Whitney U = 1.8, n_1 _= 22, n_2 _= 20, P > 0.5 two-tailed).

Since both LD and SD groups were vernalized under SD light conditions at 4°C, a control group of LD plants was shifted to SD conditions but maintained at 20°C in order to rule out a shift in daylength as initiating flowering. Of the plants that were not vernalized, 78% (n = 14) did not flower after 85 days "post-vernalization", indicating that a shift in daylength alone does not promote flowering and affirming the importance of vernalization for floral induction in *A. formosa *(Figure 2A). Importantly, this study has also revealed that there is some natural genetic variation in response to vernalization as 22% (n = 4) of the non-vernalized plants did flower without vernalization, but the range over which these plants flowered was much wider than the vernalized plants. In addition, we have occasionally observed rare individuals grown from wild collected seed that flower very early, before plants reach the size when competence to respond to vernalization is thought to begin [39].

### Morphological dynamics of vernalization response

Given that *A. formosa *showed such a strong response to vernalization treatment, we decided to study the SAM before, during, and immediately after vernalization to see when the meristem transitions from a vegetative to an inflorescence meristem. Chiefly, we wanted to address whether the meristem showed developmental responses during vernalization or whether these changes occurred after the plants were removed from cold treatment. Plants were grown in LD at 20°C. Upon reaching 12 to 15 leaves, plants were moved to vernalization conditions, SD at 4°C. Apical meristems were collected and prepared for histology at five time-points, just prior to vernalization, two weeks into vernalization, six weeks into vernalization, eight weeks into vernalization (also the last day of vernalization), and one-week post vernalization. Five plants were examined at each time point and the meristem morphologies were highly consistent across all plants sectioned at a given time point.

*Aquilegia formosa *produces a cymose inflorescence in which the SAM is transformed into an inflorescence meristem, produces two lateral bracts each with axillary meristems and is then consumed by a terminal flower. The axillary meristems in turn repeat this developmental pattern. Prior to vernalization, the meristem consists of a small round dome flanked by leaf primordia and older leaves (Figure 2B). The SAM and leaf insertion points are all on the same horizontal plane, indicating very little internodal elongation. After two weeks of vernalization, there may be some subtle elongation of the meristem but by six weeks into vernalization, the meristem has clearly become vertically elongated (Figure 2B). Internodal elongation separates the most recently produced lateral organs from the rosette leaves and darkly stained meristems appear to be developing in the axils of these leaves, which is not observed at earlier stages (Figure 2B). By eight weeks, the terminal meristem has clearly transitioned to floral identity and sepals are beginning to develop. Well-defined axillary meristems are present, which may have inflorescence or floral identity (Figure 2B). One week following vernalization, the sepals of the terminal flower have elongated to nearly the height of the floral meristem and petal and stamen primordia are present. The axillary floral meristems have defined sepal primordia (Figure 2B). Axillary meristems subsequently produced by the secondary meristems are present in adjacent sections (data not shown). Thus, while photoperiod may have some impact on growth rate after vernalization, the meristem actually transitions to flowering during cold treatment.

### Flowering time genetics

In order to identify *Aquilegia *homologs of the Arabidopsis flowering time loci, we used complementary approaches of querying the *Aquilegia *DFCI Gene Index along with targeted amplification of genes of interest. A summary of *Aquilegia *homologs is found in Table 1.

**Table 1:** Arabidopsis and *Aquilegia *gene names and accession numbers

Arabidopsis gene	Locus ID	*Aquilegia *gene	Accession number
*GI*	AT1G22770	*AqGI*	HQ173334
*CO*	AT5G15840	*AqCO*	HQ173331
*FT*	AT1G65480	*AqFT*	HQ173333
*PHYA*	AT1G09570	*AqPHYA*	GQ471030
*PHYB*	AT2G18790	*AqPHYB*	GQ471031
*CRY1*	AT4G08920	*AqCRY1*	DR915774
*CRY2*	AT1G04400	*AqCRY2*	DT748827
*SOC1*	AT2G45660	*AqSOC1*	HQ173336
*AGL24*	AT4G24540	*AqAGL24.1*	HQ173338
		*AqAGL24.2*	HQ173339
*LFY*	AT5G61850	*AqLFY*	HQ173335
*FUL*	AT5G60910	*AqFL1*	DT758909
		*AqFL2*	HQ322376
*FRI*	AT4G00650	*AqFRI*	HQ173332

### Floral pathway integrators

MADS-domain containing genes, defined by the conserved *M*CM1/*A*GAMOUS/*D*EFICIENS/*S*RF domain, form a large and important family of transcription factors in plants, including the FPI genes *SOC1*, *AGL24*, and *AP1*. The evolution of this family is very complex, with the presence of numerous lineage-specific duplication events that often make it impossible to assign strict orthology to homologous genes from different taxa [42]. Our first step was to classify all of the cloned and/or annotated *Aquilegia *type II MADS box genes in the context of a broad phylogenetic analysis (See Additional File 2: Figure 1). This revealed two representatives of the *AP1/FUL *lineage, two of *StMADS11/AGL24/SVP *and one of *TM3/SOC1 *(Table 1). The two *Aquilegia FUL*-like homologs appear to have been derived from a previously characterized Ranunculales-specific duplication event and are, therefore, ancestral to all three of the core eudicot paralogous lineages containing Arabidopsis *AP1*, *FUL *and *AGL79 *[43]. Phylogenetic analysis of the two *AGL24*-like genes, *AqAGL24.1 *and *AqAGL24.2*, in the context of a larger nucleotide dataset for the *StMADS11 *clade indicates that these loci diverged prior to a duplication event that led to the formation of *AGL24 *and *SVP *lineages, however there is little support for the topology (See Additional File 2: Figure 2A). Thus, determining strict orthology between the *Aquilegia AGL24*-like genes and Arabidopsis homologs is not currently possible. The single representative of the *TM3 *clade identified so far, *AqSOC1*, belongs to the lineage of eudicot genes that also includes Arabidopsis *SOC1 *to the exclusion of the other Arabidopsis genes in the *TM3 *clade (See Additional File 2: Figure 2B).

In addition to *FT*, there are five other phosphatidylethanolamine binding protein (PEBP) genes in the Arabidopsis genome: *TERMINAL FLOWER *(*TFL*), *TWIN SISTER OF FT *(*TSF*), *BROTHER OF FT AND TFL *(*BFT*), *MOTHER OF FT AND TFL *(*MFT*), and *ACT *(*Arabidopsis thaliana CENTRORADIALIS*). We used a targeted approach to identify homologs of *FT *and *TFL *in *Aquilegia*. Although *TFL *is primarily thought of as a regulator of inflorescence structure, *tfl *mutants also show early flowering, suggesting an additional role in flowering time [44]. We also identified a third PEBP-like gene in the *Aquilegia *DFCI Gene Index. Phylogenetic analysis confirms that the loci we obtained by targeted cloning represent homologs of *FT *and *TFL*, and are therefore termed *AqFT *and *AqTFL*, respectively (Additional File 2: Figure 3). Although the presence in Arabidopsis of the recent Brassicaceae-specific *FT *paralog *TSF *rules out the possibility of strict orthology between *FT *and *AqFT*, the *AqFT *locus is clearly a member of the *FT *subfamily and is more closely related to *FT *than any of the other PEBP family members. The other PEBP gene identified in the EST database comes out in a clade with Arabidopsis *MFT *and we will refer to this locus as *AqMFT*.

**Figure 3:**
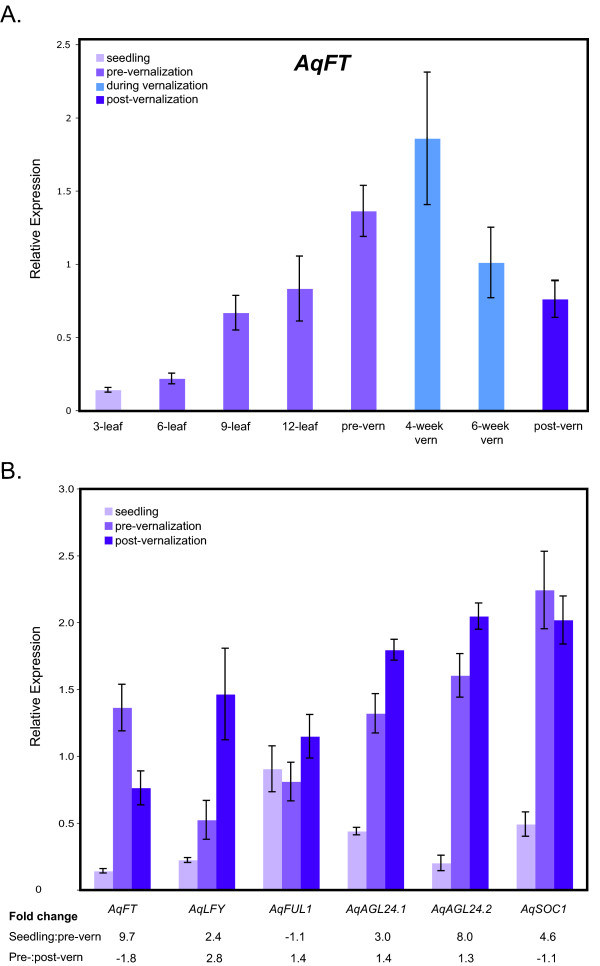
**Gene expression levels of *Aquilegia *flowering time homologs over the course of development**. **A**. *AqFT *expression levels over the course of pre-vernalization development followed by during vernalization and post-vernalization time points, +/- the standard error. **B**. All FPI homologs were assayed for relative expression at three time points: seedlings, pre-vernalization (pre-vern) 12 to 15 leaf plants, and seven-day post-vernalization (post-vern) plants. Plant apices and young leaves were collected for RNA isolation 7.5 h after dawn in LD. Relative mean expression of genes from six plants at each developmental time point, +/- the standard error. Fold change between the seedling and the pre-vernalization and the pre-vernalization and the post-vernalization values is presented for each gene.

*LFY *is not part of a complex gene family and homologs are most often represented by a single copy in angiosperms, the primary exceptions being taxa that have experienced relatively recent genome duplications [45-47]. Consistent with this, we isolated one *LFY *homolog from *Aquilegia *with 59% nucleotide identity to Arabidopsis *LFY *and phylogenetic analysis clearly indicates that it is the *Aquilegia LFY *ortholog, *AqLFY *(See Additional File 2: Figure 4).

**Figure 4:**
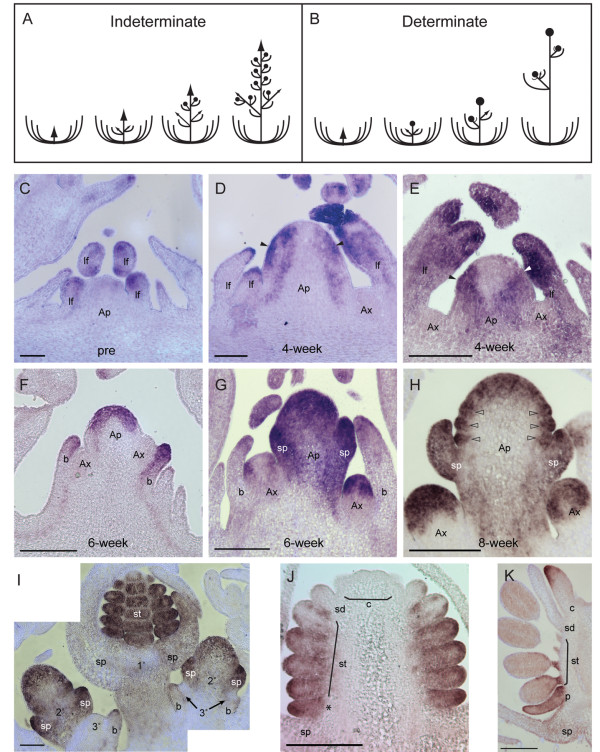
**Schematic of a racemose inflorescence and an *Aquilegia *dichasial cymose inflorescence and *AqLFY in situ *expression patterns**. In a racemose inflorescence **(A)**, such as those of Arabidopsis and *Antirrhinum*, the apical meristem remains indeterminate. Secondary indeterminate inflorescences (coflorescences) may form in the axillary meristems of the first few nodes, followed by the production of determinate flowers in the subsequent axillary meristems. In the *Aquilegia *cymose inflorescence **(B)**, the apical meristem produces two bracts with axillary meristems and then terminates in a flower. Secondary inflorescences develop from these axillary meristems. The secondary inflorescences also terminate in a flower with tertiary inflorescences developing in the axils of these flowers. This process repeats indefinitely. Expression of *AqLFY *in a pre-vernalization apical meristem **(C)**, apical meristems four weeks into vernalization **(D, E)**, apical meristems six weeks into vernalization **(F, G)**, an apical meristem eight weeks into vernalization **(H)**, a later inflorescence shown with three hierarchical degrees of floral and inflorescence meristems **(I) **and developing flowers at stage 7 **(J) **and stage 9 **(K)**. Ap = apical meristem, Ax = axillary meristem, lf = leaf, b = bract, sp = sepal, p = petal, st = stamen, sd = staminodia, c = carpel. Solid arrowheads indicate location of incipient bract, open arrowheads indicate petal and stamen primordia. Asterisk in J: Due to the floral architecture of *Aquilegia*, the basal-most primordium in a vertical rank of organs may be a petal or a stamen. These are indistinguishable at early stages so we cannot differentiate between the two possibilities in this section. Scale bar = 50 μm C-D, I; 100 μm E-H; 200 μm J-K. sp = sepal,

### Photoperiod and circadian regulation

*PHYA *and *PHYB *as well as *CRY1 *and *CRY2 *orthologs from *Aquilegia *were identified (See Additional File 2: Figure 5). A search of the *Aquilegia *DFCI Gene Index did not reveal any homologs to the circadian clock gene *TOC1 *but sequence fragments with similarity to *CCA1 *and *LHY *were obtained (DT742775, TC22162). These were not pursued further since the core circadian clock is not the focus of this study. We also recovered 5' and 3' ends of an apparent *Aquilegia GI *ortholog, termed *AqGI*, and these regions were used to obtain the complete cDNA sequence using RT-PCR. Similar to *LFY*, the lineage evolution of *GI *is rather straightforward and phylogenetic analyses support our identification of *AqGI *as orthologous to Arabidopsis *GI *(See Additional File 2: Figure 6).

**Figure 5:**
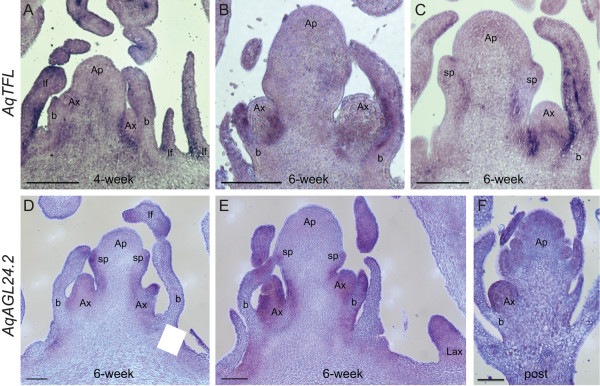
***AqTFL1 *and *AqAGL24.2 in situ *expression patterns**. *AqTFL1 *expression in four-week **(A) **and six-week **(B, C) **vernalized apices. *AqAGL24.2 *expression in six-week **(D, E) **vernalized apices and in a higher order post vernalized inflorescence **(F)**. Ap = apical meristem, Ax = axillary meristem, Lax = leaf axillary meristem, lf = leaf, b = bract, sp = sepal, open arrowheads indicate petal and stamen primordia, arrows indicate *AqAGL24 *expression in leaves. Scale bar = 100 μm A-C; 50 μm D-F.

**Figure 6:**
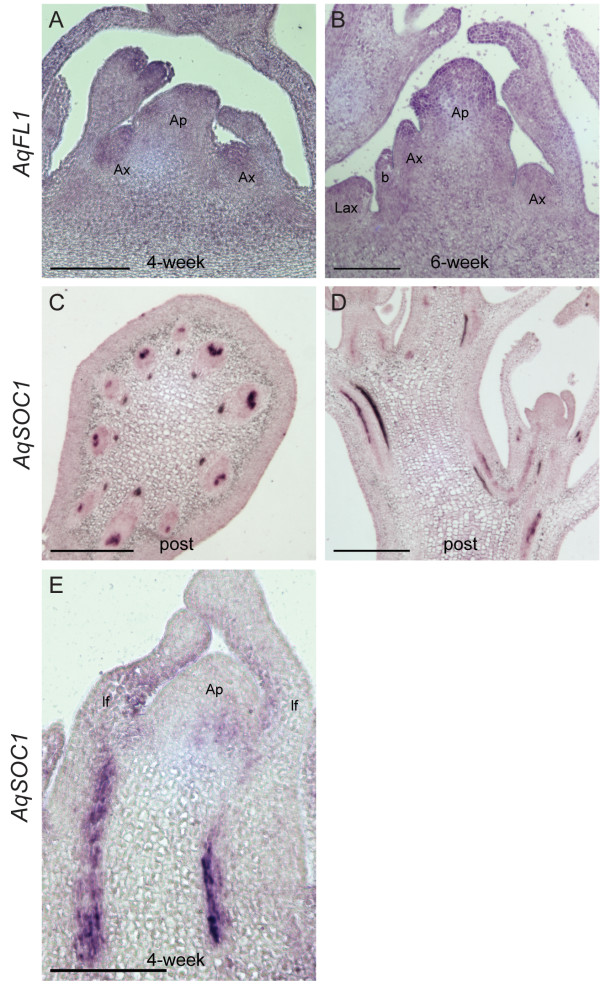
***AqFL1 *and *AqSOC1 in situ *expression patterns**. *AqFL1 *expression in four-week **(A) **and six-week **(B) **vernalized apices. *AqSOC1 *expression in a transverse section of a post-vernalization peduncle **(C)**, a longitudinal section of a post-vernalization inflorescence **(D)**, and a four-week vernalized apex **(E)**. Ap = apical meristem, Ax = axillary meristem, b = bract, Lax = leaf axillary meristem, lf = leaf. Scale bar = 100 μm A-C; Scale bar = 200 μm D, E.

On the other end of the spectrum, there are 17 genes in the Arabidopsis genome that show some similarity to *CO *based on the presence of conserved B-box and CCT domains, including two very recent paralogs of *CO*, *COL1 *and *COL2 *[48]. Genomic searches of other taxa such as rice and barley have likewise revealed the presence of multiple *CO*-like genes, indicating a complex history for the gene family. It can thus be difficult to determine orthology between *CO *homologs from different taxa. A potential *Aquilegia CO *ortholog was obtained by targeted amplification and identical fragments were found in the *Aquilegia *DFCI Gene Index. In addition to *AqCO*, two other *CO*-like genes, *A. formosa TC932 *and *A. formosa DR918*, were identified from the DFCI Gene Index. Phylogenetic analysis revealed that the product of the targeted cloning falls within a highly supported clade that also includes Arabidopsis *CO *and *Oryza Hd1*, with monocot and core eudicot homologs each forming monophyletic groups and *AqCO *sister to the core eudicot homologs (See Additional File 2: Figure 7). This indicates that *AqCO *is a close homolog of Arabidopsis *CO *but the *Aquilegia *locus is also equally related to the recent Arabidopsis paralogs *COL1 *and *COL2*.

### Vernalization and autonomous pathways

We did not have an *a priori *expectation to recover homologs of the Arabidopsis vernalization pathway loci since it appears that this program is less conserved than other flowering time pathways [49]. Even though *FLC *plays a critical role in vernalization response in Arabidopsis, it is well-known that homologs of *FLC *have not been identified outside the core eudicots [50,51]. Despite recovering many MADS-box genes in the *Aquilegia *DFCI Gene Index, we were unable to identify loci belonging to the *FLC *subfamily (See Additional File 2: Figure 1). *Aquilegia *loci involved in chromatin remodeling are being annotated in the context of another project and so were not pursued in this study, but two genes showing similarity to *FRI *were found. Phylogenetic analysis of these loci and several *FRI*-like genes from various taxa reveal that one of the genes, termed *AqFRI*, is a member of the clade that also contains *FRI *from Arabidopsis (See Additional File 2: Figure 8).

Sequence fragments with homology to the autonomous pathway genes *FCA *and *FY *were identified in the *Aquilegia *EST database (*AqFCA*: TC28874, *AqFY*: TC32137; L. Holappa and E. Kramer pers. comm.). Given their pleiotropic roles in RNA processing and chromatin remodeling [52,53], it is not surprising that there are similar genes present in *Aquilegia*, but whether they will have a role specific to flowering time or will more generally effect plant developmental and regulatory processes is unknown.

### Regulatory pathways controlling *AqFT*

In several taxa including Arabidopsis, rice, poplar, and cucurbit, there is evidence that the regulation of *FT *homologs in response to environmental conditions is a key trigger controlling the induction of flowering [54-58]. In Arabidopsis and grasses, homologs of *GI*, *CO*, and *FT *form a regulatory module to promote flowering under distinct photoperiod conditions (reviewed [29]). Additionally, in some Arabidopsis ecotypes, vernalization is critical to the alleviation of factors that repress *FT *expression prior to flowering. To gain an understanding for whether and how *AqFT *is regulated by environmental conditions, we analyzed its expression in the context of the proposed *GI-CO-FT *photoperiod regulon as well as in response to vernalization.

First, we assayed whether *AqFT *showed any diurnal expression variation in reference to *AqGI *and *AqCO*. Plants were entrained in either LD or SD immediately upon removal from vernalization. Ten days later, leaf samples were taken every four hours starting at presumptive dawn (0 h) and quantitative RT-PCR was used to examine the relative expression of *AqGI*, *AqCO*, and *AqFT*. Expression levels of these genes were normalized to the expression of the *Aquilegia *homolog of *isopentyl pyrophosphate:dimethylallyl pyrophosphate isomerase2 (AqIPP2) *at each time point [59]. Values presented for each time-point are relative to normalized values of the gene of interest from a control sample that consisted of a mix of all cDNAs used in the experiment.

*AqGI *expression was very similar to what has been observed in other systems with a narrow SD peak at 8 h and a broader LD peak between 8 to 12 h (See Additional File 1, Figure 2). Under both conditions, *AqCO *reaches its peak around presumptive dawn (0 h) then is quickly down-regulated, but it shows a much stronger diurnal signal in SD than LD (See Additional File 2, Figure 2). There is an approximately 12X difference in *AqCO *expression between the low and high points in SD, while in LD it only varies approximately 3X. In contrast to Arabidopsis, it appears that *AqCO *baseline transcription is generally quite high in *Aquilegia*. We had no difficulty detecting a signal from *AqCO *in Northern blot analyses (data not shown), whereas in Arabidopsis, PCR amplification of *CO *is necessary before expression can be detected by Northern [30]. In addition, when running standard curves for quantitative RT-PCR, *AqCO *could always be detected several cycles prior to *AqGI*, *AqFT*, and *AqIPP2 *(data not shown).

While *AqGI *and *AqCO *show diurnal regulation in both SD and LD, the expression pattern of *AqFT *is more complex (See Additional File 2 Figure 2). In SD, *AqFT *has near zero relative expression at all time points except for at 8 h, where there is a spike in expression. Although the amplitude of the spike is variable, all samples show a distinct uptick relative to the 4 h time point (data not shown). The expression pattern of *AqFT *in LD conditions is not clearly suggestive of diurnal regulation. Instead, it appears that *AqFT *expression in LD is expressed at relatively high levels throughout the day with the highest (16 h) and lowest (4 h) points of mRNA abundance differing only approximately 3X. Generally, *AqFT *exhibits higher overall expression levels in LD than in SD (See Additional File 2, Figure 2). In summary, although all three loci show evidence of diurnal regulation, their patterns are not clearly related to one another and, possibly consistent with a day-neutral condition, *AqFT *is expressed under both SD and LD conditions.

The diurnal experiments were all conducted in post-vernalization plants so the question remained as to how vernalization might regulate *AqFT *expression. Therefore, we used qRT-PCR to examine *AqFT *expression in early seedlings though vernalization to post-vernalization growth. Plants were grown under LD conditions and all samples were taken at 7 h post dawn. These data show that *AqFT *expression gradually increases during vegetative development, reaching a peak four weeks into vernalization before decreasing again (Figure 3A).

### Floral pathway integrator expression patterns

Most of the Arabidopsis FPIs show moderate to strong up-regulation coincident with the floral transition [19,54,60]. By characterizing the expression of *Aquilegia *FPI homologs before and after vernalization, we hoped to gain insight into whether increased expression of these loci is similarly associated with flowering and inflorescence development in *A. formosa*. Quantitative RT-PCR was used to assess the expression levels of *AqAGL24.1*, *AqAGL24.2*, *AqSOC1*, *AqLFY *and *AqFL1 *in unvernalized plants at both seedling and 12 to 15 leaf stages and in plants following an eight week vernalization period (Figure 3B). We did not pursue expression studies of the other *Aquilegia FUL*-like gene, *AqFL2*, as preliminary studies indicate that it is only expressed at very low levels in late stage flowers (A. Litt pers. comm.). *AqLFY*, *AqAGL24.1*, and *AqAGL24.2 *all show statistically significant increases in expression (Student's *t*-test probability <0.05) following vernalization, with the approximately three-fold increase in *AqLFY *being the most dramatic. *AqFL1 *expression shows a slight, statistically non-significant increase following vernalization while *AqSOC1 *shows a slight non-significant decrease in expression following vernalization. If these results are examined in the context of the seedling expression patterns, we see that, with the exception of *AqFL1*, the FPI homologs are expressed at very low levels in three-leaf seedlings relative to pre-vernalization plants at the 12 to 15 leaf stage (Figure 3B). The most dramatic expression increases between the three-leaf and pre-vernalization stages is seen in *AqAGL24.2*, which rises approximately eight-fold (compare with *AqFT*, shown for reference, which also shows a strong induction of approximately ten-fold). *AqLFY*, *AqAGL24.1*, and *AqSOC1 *show moderate transcription increases in the range of two- to five-fold. Unlike the other FPI homologs, expression of *AqFL1 *commences early and is relatively stable at the various developmental time points we examined.

In Arabidopsis, the Floral Pathway Integrators control the transition to flowering in part by conferring inflorescence and/or floral identity to meristems. Because inflorescence meristems are by definition indeterminate while floral meristems are determinate, the demarcation pattern of inflorescence and floral meristems also contributes to inflorescence architecture. Therefore, along with their role in flowering time, the FPIs are also crucial to inflorescence architecture. In Arabidopsis, a plant with an indeterminate inflorescence similar to that shown in Figure 4A, the apical meristem maintains inflorescence meristem identity throughout the period of flowering, producing lateral determinate floral meristems. *Aquilegia *has a determinate cymose inflorescence, depicted in Figure 4B, in which inflorescence identity is a transient state rather than a persistent one. Every inflorescence meristem, starting with the primary apical meristem, produces two lateral bracts each with its own axillary inflorescence meristem, and then is itself transformed into a determinate floral meristem. The axillary meristems reiterate this pattern repeatedly during flowering. To gain a better understanding for how FPI homologs may be functioning to both promote flowering and determine inflorescence architecture during the floral transition in *A. formosa*, we further analyzed their spatio-temporal expression patterns before, during, and after vernalization using *in situ *hybridization. To aid in descriptions of floral development, *Aquilegia *floral stages are presented in reference to Arabidopsis floral stages in Additional File 3, Table 1.

The expression of *LFY *homologs is indicative of floral meristem identity in many taxa [61] and, consistent with this, qRT-PCR data demonstrate that *AqLFY *is strongly induced following vernalization and the floral transition (Figure 3B). *In situ *hybridization of the *AqLFY *transcript shows that prior to vernalization, the gene is expressed in leaf primordia and young leaves as well as at the margins of older leaves, but is not expressed in the SAM or in axillary meristems (Figure 4C). While there is some variation in the developmental stages of the four- and six-week vernalized apices, generally by four weeks into vernalization the apical meristem has begun to elongate and *AqLFY *is detected in the flanks of the meristem while being notably absent from the central zone and incipient axillary meristems (Figure 4 D, E). This expression appears to be concentrated in the regions that will give rise to the opposite bracts subtending the first axillary meristems of the inflorescence (Figure 4 D, E). Figure 4F represents a developmental stage that typically occurs between four and six weeks of vernalization, at which point the first axillary meristems on the inflorescence are well-formed. At this stage, *AqLFY *is now seen throughout the central region of the apical meristem and in the bracts subtending the axillary meristems, but is not detected in the axillary meristems themselves (Figure 4F). In six-week vernalization apices that are at a slightly later developmental stage, *AqLFY *is strongly expressed throughout what is now a stage three terminal flower, including the differentiating sepal primordia (Figure 4G). The axillary meristems have further developed and exhibit *AqLFY *expression patterns similar to those of four-week apical meristems, indicating that they are reiterating the transition to inflorescence identity and then floral identity (Figure 4G Eight weeks into vernalization, the terminal flower is at approximately stage 5, with well-differentiated sepals and initiating petal and stamen primordia. *AqLFY *is expressed in the sepals and the peripheral regions of the meristem containing the petal, stamen and incipient staminodium primordia, but is no longer expressed in the central region of the meristem (Figure 4H). Looking at later stages of the inflorescence, *AqLFY *expression can be simultaneously visualized in three hierarchically-staged meristems (Figure 4I). In the primary meristem, a stage 8 flower with well-defined stamens, *AqLFY *is detected strongly in the stamens. The secondary meristems are at earlier stages of floral development, approximately stage 3 and 4, and *AqLFY *is present throughout the floral meristem including the developing sepals. *AqLFY *is conspicuously absent from the incipient tertiary meristems, which are young inflorescence meristems, but it is detected in the bracts subtending these meristems.

As flowers continue to develop, *AqLFY *is gradually turned off in the developing organs in a basipetal fashion (Figure 4J, K). *AqLFY *appears to decline in the apex before carpel primordia begin to initiate and is then progressively excluded from the staminodia (sterile organs found in a whorl between the carpels and stamens in *Aquilegia*) followed by the most apical stamens (Figure 4J). In late stage flowers, *AqLFY *is detected in the proximal filaments of the most basal stamens and throughout the petal but is also detected in the tips of developing carpels, indicating reactivation at late stages (Figure 4K).

In contrast to the *LFY*-like genes, *TFL1 *homologs have been linked to the maintenance of indeterminacy in inflorescence meristems [62,63]. We studied *AqTFL1 *expression with the hypothesis that the locus would be expressed in inflorescence meristems complementary to *AqLFY*. Obvious staining was not detected in pre-vernalized apices, although signal can be seen in leaves, particularly the petiole (data not shown). In four- and six-week vernalized plants, weak to moderate expression is detected in a zone subtending the axillary meristems, as if demarcating a boundary between the axillary meristem and the primary apical meristem (Figure 5A-C). Transcripts were also detected on the adaxial sides of bracts subtending axillary meristems (Figure 5A-C). We were never able to recover mutually exclusive expression patterns of *AqLFY *and *AqTFL1*.

Like *TFL1*, *AGL24 *contributes to inflorescence meristem identity in Arabidopsis [20]. Of the two *Aquilegia AGL24 *homologs, we had greater success with the *AqAGL24.2 *probe. The strongest *AqAGL24.2 *expression was detected in six-week vernalization apices (Figure 5 D, E). In these apices, *AqAGL24.2 *expression is concentrated in axillary meristems and in the young bracts subtending these meristems (Figure 5 D, E). At this stage, the sepal primordia are barely beginning to form on the terminal flower. Weak expression is detected in the edges of these sepal primordia and continues in the flanks of the pedicel and in the flanks of the peduncle, however, expression is not detected throughout the apical dome of the terminal flower at this stage (Figure 5 D, E). Interestingly, expression is detected in the axillary meristems of older leaves, in meristems where *AqLFY *expression was not detected (labelled Lax in Figure 5E). In older flowers, there may be some *AqAGL24.2 *expression in developing sepals and early petal and stamen primordia, but expression is not strong enough to be definitive (Figure 5F). *AqAGL24.2 *expression can also be seen in lateral portions of young leaves, similar to *AqLFY *(Figure 5D).

Spatial expression of *AqFL1 *was difficult to assess. In several sections, increased staining appears concentrated in the meristematic regions of the apex. Similarly, staining appears somewhat concentrated in the axillary meristems and in leaf tips in four-week vernalized apices (Figure 6A). In some early stage six-week vernalized apices, *AqFL1 *expression is more strongly detected throughout the dome of the apical floral meristem, including in the bract primordia and in the tips of young leaves and leaflets, however, strong expression was not detected consistently (Figure 6B). The qRT-PCR data show that *AqFL1 *is expressed at consistent levels at all of the developmental stages we tested, including in very young seedlings (Figure 3B). Therefore, it may be that *AqFL1 *has constitutive weak to moderate expression relative to the background and does not have specific expression in inflorescence or floral meristems.

Contrary to *SOC1 *in Arabidopsis, *AqSOC1 *shows strong and consistent expression in vasculature throughout the aerial portions of the plant (roots were not examined), including leaf and stem vasculature. Vascular expression was easily detected at all time points examined (pre-, during, and post-vernalization) and does not appear to change during vernalization (Figure 6C-E), consistent with qRT-PCR data (Figure 3B). Weak expression was also detected in the central region of some apical meristems (Figure 6E).

## Discussion

*Aquilegia *is poised to become an important new model system for exploring many aspects of plant biology from genetics, genomics, and development to evolution and ecology. Because of the unique nature of the *Aquilegia *species complex and the position of *Aquilegia *within the angiosperm phylogeny, studying a trait such as flowering time can provide insight into both macro- and microevolutionary questions. In addition, a more comprehensive understanding of flowering control in *Aquilegia *will help researchers manipulate this important trait, potentially reducing generation time and thereby increasing efficiency of further genetic research in the genus. Our data show that vernalization is a key regulator of flowering in these accessions of *A. formosa *and suggest that photoperiod has little or no role. Nevertheless, we have discovered that *Aquilegia *has homologs of loci known to function in the photoperiod pathway along with those from the FPI, vernalization, and autonomous flowering programs.

### Response to environmental signals

Plants have evolved several genetic mechanisms to judge seasonality and determine the best time to initiate reproductive growth. The ability to measure day length and temperature is key to establishing seasonally controlled responses. While taxa such as Arabidopsis use both photoperiod and vernalization to regulate flowering, in the high elevation *Aquilegia formosa *populations that we sampled, it appears that temperature has taken a priority over photoperiod. Somewhat unexpectedly, we found that the floral transition actually occurs during the vernalization period (Figure 2B). In this case vernalization was under SD conditions, but exposure to SD alone without a corresponding drop in temperature does not induce a strong flowering response (Figure 2A). Although the statistical measures of flowering time in SD versus LD vary in significance depending on the inclusion of three SD plants that did not flower, we believe that the measured differences in flowering time between LD and SD are more likely attributable to differences in the rate of inflorescence elongation in SD versus LD, not in the timing of the transition of the apical meristem from a vegetative meristem to an inflorescence meristem. This conclusion is strongly supported by the finding that the floral transition occurs during vernalization. We did not test the effect of vernalizing using LD conditions, in part because the natural phenology of these plants would always have vernalization occurring during fall or winter when days are short. It is also important to note that previous studies of *Aquilegia *floral induction response did test vernalization under LD or even constant light and still saw strong acceleration of flowering by cold treatment [64], so it is reasonable to conclude that SD is neither sufficient nor required for floral induction in *A. formosa*.

This is not to say that *A. formosa *does not show developmental responses to SD. In comparison to those grown in SD, LD plants generally produced leaves that were borne on much longer petioles and each of the leaf blades expanded to a greater degree, effectively producing larger plants (See Additional File 1: Figure 1). In our study, three of the SD plants had not flowered 85 days after vernalization and several of the SD plants only produced one to three flowers on an inflorescence that showed little elongation (data not shown). A possible explanation for this is that although these SD plants had 12 to 15 leaves prior to vernalization, they were not physiologically capable of responding to induction or producing robust inflorescences, possibly due to insufficient energy stores or lesser biomass. This difference in inflorescence size was not observed in one of our pilot studies where all plants were grown in LD conditions prior to vernalization and were shifted into different LD and SD conditions only after vernalization (data not shown). Consistent with this observation, it has been reported that in some *Aquilegia *species, inflorescence height is affected by both the duration of vernalization and photoperiod [64]. Thus, although day length alone is not the primary environmental trigger promoting flowering, growth in long days may be important for increasing plant biomass prior to vernalization, insuring that plants are competent to flower and capable of producing larger inflorescences that bear more flowers following vernalization. In addition, we have uncovered evidence for natural variation in *Aquilegia*'s requirement for vernalization, much like what has been observed for Arabidopsis [33-35], which will require further study.

### Flowering time gene homologs

We have identified a diverse set of flowering time homologs in *Aquilegia formosa*. These genes provide a framework for studying the genetic basis of variation in flowering time control between populations of *A. formosa *and across the *Aquilegia *genus. For some loci involved in the regulation of flowering time in Arabidopsis, we can be fairly confident that we have identified the orthologous gene from *Aquilegia. GI *and *LFY*, for example, usually exist as single copy genes [45,47]. Other Arabidopsis genes involved in flowering time, however, belong to gene families with much more complicated evolutionary histories, making it difficult to assign homology. In comparing *Aquilegia *to Arabidopsis, at least three genome duplications are spanned: one specific to the Ranunculales, one at the base of the core eudicots, and one specific to the Brassicaceae [4,65]. Similarly, the grass model systems have experienced their own independent genome duplications [66]. These events make it impossible to assign strict orthology between many of the *Aquilegia *MADS-box genes and the homologous Arabidopsis or grass flowering time genes, although we have recovered homologs of the critical *AP1/FUL/VRN1*, *AGL24/SVP/VRT2 *and *SOC1/OsMADS50 *lineages. Likewise, *CO *has two closely related paralogs in Arabidopsis, *COL1 *and *COL2*, and within the PEBP gene family, there is a closely related recent paralog of *FT *in Arabidopsis, *TSF*. Regardless of whether or not simple orthology can be identified between Arabidopsis, grass and *Aquilegia *genes, studying the identified *Aquilegia *homologs will help us to understand the evolution of function across these gene families. One notable exception to this list is a clear homolog to *FLC*. Studies in the grasses have already found that vernalization response is not controlled by *FLC *homologs in those systems [49], so it appears that vernalization pathways across the angiosperms are not composed of homologous components. It will be necessary, therefore, to identify new candidates for the regulation of vernalization response in *Aquilegia*.

### *AqFT *expression and the floral transition

Extensive research into the photoperiodic control of flowering time in Arabidopsis has highlighted the importance of *GI*, *CO*, and *FT*. Mutations in any of these genes result in delayed flowering in inductive long day conditions relative to wild type. These loci have been placed in a genetic pathway whereby *GI *positively regulates *CO*, which in turn positively regulates *FT*. Elements of this pathway appear to be conserved across angiosperms with different photoperiod responses, including the SD-flowering monocot rice [67]. However, this study is the first examination of the diurnal regulation of these genes in a potentially day neutral plant. Overall, the expression of *AqGI *appears to be conserved relative to diverse angiosperm orthologs [67-70], perhaps indicating a conserved role in the central oscillator of the circadian clock [68]. Contrary to *AqGI*, the diurnal expression patterns of *AqCO *and *AqFT *are not conserved between *Aquilegia *and Arabidopsis, particularly in regard to the expression of the loci relative to one another. These findings suggest that the GI-CO-FT regulatory module is not functioning in the same manner in *Aquilegia *but that does not mean that *AqFT *plays no role in floral promotion. In the day neutral species *Solanum tuberosum*, ectopic *CO *expression does not promote flowering but the *FT *homolog is still known to induce floral transition in the closely related *Solanum lycopersicon *[71,72]. In this regard, it is important to note that AqFT shows 83% amino acid similarity with Arabidopsis FT, including the functionally critical position 83 tyrosine [73], so there is every reason to believe that the proteins are biochemically equivalent.

We were surprised to find that *AqFT *is actually expressed at significant levels before vernalization and the floral transition. This expression increases gradually throughout vegetative development, reaching a peak at four weeks into vernalization, a period that is associated with the floral transition based on histology and *AqLFY *expression. The variation that exists between biological samples at the four-week vernalization point may reflect some variation among individuals in the exact timing of the transition to flowering (Figure 3A). The gradual up-regulation of *AqFT *is reminiscent of a similar pattern observed in *Populus*, where the *FT *homolog *PtFT1 *has been shown to promote flowering in a dosage-dependent manner [65]. Thus, although *FT *homologs have been shown to promote flowering in many dicot and monocot systems, a perfect correlation between their expression and floral induction is not always observed. This suggests that the exact mechanisms by which *FT *homologs are regulated or specifically achieve their function can vary. Along these lines, one possible explanation for the upward trend in *AqFT *expression is that the gene functions on a very fine threshold of expression such that the difference between the pre-vernalization and 4-week vernalization expression levels is enough to initiate flowering. Another possibility is that vernalization regulates flowering downstream or in parallel to *AqFT *function, including regulation of AqFT protein localization [74], which appears to be the primary FT regulatory mechanism in regard to photoperiod in the Cucurbits [58]. Distinguishing between these possibilities will require the identification of *AqFT *loss-of-function mutants or the development of *Aquilegia *transgenics since VIGS, which tends to produce silenced sectors, is not well suited to the analysis of non-cell autonomous gene functions.

### FPI homolog expression in response to vernalization

In studying the expression of *AqCO *and the selected *Aquilegia *FPI homologs before and after vernalization, we hoped to identify loci that are differentially expressed in response to vernalization with the thought that such genes may function in either repression or induction of flowering. In particular, the Arabidopsis FPIs are strongly up-regulated in inflorescence and floral meristems and thus if their functions are conserved with the *Aquilegia *homologs, we would expect them to be similarly up-regulated following vernalization in *A. formosa*. While several of the FPI homologs show statistically significant increases in expression after vernalization, these differences are rather subtle. *AqLFY *is the only locus to show an expression increase of greater than two-fold following vernalization treatment, which is consistent with *AqLFY *playing a conserved role in initiating the floral transition downstream of vernalization. Although *AqLFY *is strongly expressed pre-vernalization in developing compound leaves, expression in meristems is only observed after four to six weeks of vernalization and, based on other histological features, appears to coincide with the establishment of floral meristem identity. Strong expression of *AqLFY *in young leaves and leaflets may indicate a role for the gene in the development of *Aquilegia*'s compound leaves, which has been shown for *LFY *homologs in legumes and suggested for homologs in *Eschscholtzia *[75-77].

The functional antagonist of *LFY *in Arabidopsis is the inflorescence identity gene *TFL1*, which is expressed in a complementary manner to *LFY *[78]. The unusual expression pattern of *AqTFL1 *subtending axillary meristems does not rule out a similar function in *Aquilegia*, however, since the protein is known to function non-cell-autonomously [79]. It is, therefore, important to note that *AqTFL *expression is only associated with young axillary meristems and disappears once the meristems transition to a terminal position (Figure 5B, C), which also corresponds to the full expression of floral identity as denoted both by sepal initiation and constitutive expression of *AqLFY*.

The remaining FPI homologs - AP1, SOC1 and AGL24 - are all MADS box-containing proteins and exhibit complex interactions in Arabidopsis [21,26,80]. Adding further complication is the opposing role of *SVP*, a paralog of *AGL24 *and a floral repressor [81], although there is evidence that at some stages of floral development the AP1, AGL24 and SVP proteins may work cooperatively rather than antagonistically [80]. Neither of the *AqAGL24 *homologs shows strong up- or down-regulation relative to floral induction (which is seen respectively for Arabidopsis *AGL24 *and *SVP*) but the spatial localization of *AqAGL24.2 *is consistent with a function in inflorescence meristem identity. This raises interesting questions as to the ancestral functions of the gene lineage predating the apparent core eudicot duplication that gave rise to *AGL24 *and *SVP *- did it promote or repress flowering? However, there is simply not enough functional evidence for homologs either post- or pre-dating the duplication event to draw clear conclusions. The expression data for *AqFL1 *is rather uninformative, being fairly moderate and constitutive, both temporally and spatially. It remains possible that *AqFL1 *functions in floral meristem identity or the promotion of flowering, as has been seen for homologs in many other taxa (reviewed [82]), but this function could be delimited by differential expression of other co-factors, candidates for which include the *AqAGL24*s or *Aquilegia SEPALLATA *homologs [80,83]. The expression of *AqSOC1 *is more dramatically divergent from what is observed for *SOC1 *in Arabidopsis, which is strongly induced by flowering and is expressed in apical meristems [84]. *AqSOC1 *exhibits strong vascular staining with little or no expression in the meristem at any of the time points assessed using *in situ *hybridization, suggesting that *AqSOC1 *either plays a very different role or exerts its effect on flowering in a different manner than the Arabidopsis homolog. Work on several *SOC1*-like genes in grasses underscores the fact that while these loci are often involved in flowering time, their phylogenetic relationships are poorly resolved and their exact functions may vary considerably [85,86].

On the whole, it is important to note that the developmental expression of the floral transition is the initiation of inflorescences, which represent a dramatically different developmental program relative to vegetative development. Furthermore, the genetic basis of evolutionary shifts between determinate (cymose) and indeterminate (racemose) inflorescence structures are a major question in angiosperm evolution. In *Aquilegia*, the expression patterns for *AqLFY*, *AqAGL24*.2 and *AqTFL *suggest a hypothesis for the genetic control of meristem identity transitions during *Aquilegia *cyme development: 1) axillary meristems initially express *AqAGL24.2 *and are subtended by a zone of *AqTFL*, 2) *AqLFY *becomes expressed in the flanks of the meristem, marking the arising opposite bracts, 3) after bract initiation, *AqLFY *becomes constitutive throughout the now terminal meristem, *AqAGL24.2 *is down-regulated and the subtending *AqTFL *disappears. It remains to be determined exactly how much overlap occurs in *AqLFY *and *AqAGL24.2 *expression during the transition from inflorescence to floral identity. Also, although *AqAGL24.2 *and *AqTFL *are detected in pre-vernalization apices by qRT-PCR, no signal was detected in or around the vegetative meristems by *in situ *hybridization. This leaves open the question of whether the loci might play some role in repressing flowering before vernalization or strictly function in inflorescence identity.

## Conclusions

While we continue to gain a better understanding of the nuances of flowering time control in Arabidopsis, details on this response in other plant lineages remain scarce. *FT *homologs have been shown to play critical roles in the induction of flowering in all taxa examined so far, including LD, SD, and day neutral plants. In *A. formosa*, photoperiod has little or no effect on the timing of the floral transition and the expression pattern of *AqFT *is not strictly linked to flowering. This suggests that *AqFT *is primarily regulated at the protein level or has a necessary cofactor or target that is independently regulated by vernalization. We have confirmed that vernalization is a very important stimulus to flowering in *Aquilegia*. Unlike Arabidopsis where vernalization essentially controls the competence of specific ecotypes to respond to inductive long days, in *Aquilegia *vernalization itself is the inductive signal. Aside from a *FRI *homolog, however, we have not been able to identify any *Aquilegia *candidates for floral repressors that would function analogously to *FLC*. Downstream components of the flowering pathway, show evidence of functional conservation in the cases of *AqLFY *and *AqAGL24.2 *but not for the *AqSOC1 *and *AqFL1 *homologs. Thus, further studies of the vernalization response and floral promotion pathways in the *Aquilegia *species complex will require both functional tests and the identification of new candidate loci, a process that will provide a unique opportunity to study the independent derivation and diversification of these genetic programs.

## Methods

### Measurement of flowering time

*Aquilegia formosa *seed was collected from two localities, Lundy Canyon, CA and Stewart Creek, NV. Both localities are at approximately 38° north latitude and approximately 2,500 meters above sea level. Plants from these seeds were grown in the greenhouses at Harvard University and were then crossed to one another creating an F2 generation of hybrids from the two localities. F2 seeds were cold stratified at 4°C for eight weeks to promote germination. Seedlings were germinated and grown in either long- or short-day conditions in controlled-environment growth chambers. All plants were grown at approximately 20°C with SD plants exposed to 8 hr light/16 hr dark and LD plants exposed to 16 hr light/8 hr dark. When plants had at least 12 leaves, plants from both LD and SD growth conditions were moved into vernalization at 4°C under short day conditions. A group of plants grown in LD was shifted to SD but remained at 20°C to serve as a vernalization control. After eight weeks, plants were transferred back to their original conditions. Flowering time was measured as the number of days after removal from vernalization (or in the case of the control, the shift back to LD) at which an inflorescence was visible emerging from the apex of the sheathing leaf bases. The number of leaves present at the time of floral transition was not used as a measure of flowering time as plants grown in LD versus SD have very different leaf phenotypes, especially regarding size. Further complicating the use of leaf count for measuring flowering time is the fact that in *A. formosa*, axillary meristems commonly do not remain dormant. These meristems produce leaves and contribute to the overall photosynthetic output of the plant, but often do not produce inflorescences unless apical dominance is released.

### Histology

F2 seeds were cold stratified as in the flowering time experiment before being germinated and grown in LD at 20°C until reaching 12 to 15 leaves. Plants were vernalized at 4°C for eight weeks. Apices from five plants at each time point (before vernalization, two weeks vernalization, six weeks vernalization, eight weeks vernalization, and one week post vernalization) were collected and fixed in FAA. Following dehydration, apices were embedded in Paraplast Plus for sectioning. Sections were stained using a solution of 0.025% Alcian blue 8 GX and 0.01% Safranin O in a 0.1 M acetate buffer (pH5) (both stains, Sigma-Aldrich Corp., St. Louis, MO, USA).

### Gene cloning

In cases where BLAST ([87]) searches of the *Aquilegia *DFCI Gene Index http://compbio.dfci.harvard.edu/tgi/cgi-bin/tgi/gimain.pl?gudb=Aquilegia did not identify our genes of interest, degenerate primers were designed to conserved protein domains (Additional File 3 Table 3). Targeted loci were amplified from a mix of cDNA prepared from 10 μg of RNA isolated from pre- and post-vernalized apices and young leaves. RT-PCR fragments were cloned using the TOPO-TA Cloning Kit and TOP10 competent cells (Invitrogen, Carlsbad, CA, USA) and several clones per cloning reaction were sequenced using Big Dye technology. Specific primers were then designed for use with the SMART RACE cDNA Amplification Kit (Clontech, Mountain View, CA, USA) in order to amplify 5′ and 3′ gene ends for subsequent cloning and sequencing (Additional File 3 Table 3). In cases where gene fragments were identified from the DFCI Gene Index, specific primers were designed to obtain full-length sequences (Additional File 3: Table 3).

### Phylogenetic analysis

For all gene trees, homologs to the flowering time genes were identified for a variety of seed plant taxa by using the BLAST algorithm [87] to search the GenBank and The Institute for Genomic Research (TIGR) databases or through literature searches. With the exception of the MADS box gene phylogeny (See Additional File 2: Figure 1), phylogenetic analyses were carried out on amino acid alignments using parsimony criteria in PAUP* [88]. The MADS box tree presented in Additional File 2: Figure 1 was estimated using neighbor joining criteria as implemented by PAUP. For all datasets, amino acid sequences were initially aligned using Clustal W and then adjusted by hand using MacVector (Cary, NC, USA). For the large scale MADS-box gene phylogeny (See Additional File 2: Figure 1), the MIK domains were used, while full length sequences were used for the focused *StMADS11 *and *TM3 *phylogenies (See Additional File 2: Figure 2). For the *CO*-like genes, the alignment was edited down to the conserved B-box and CCT domains. Similarly, for the *LFY *and *FRI *alignments, only regions that could be unambiguously aligned were used in the analysis. The entire coding sequence was used for the *FT*-like, *GI*, phytochrome and cryptochrome datasets. In all cases, the tree presented is the strict consensus of all most parsimonious trees. Bootstrap analyses of 1,000 replicates were run and bootstrap values are presented at all nodes with greater than 50% support.

### Quantitative real-time PCR

For the diurnal regulation experiment, total RNA was extracted from young leaves using the RNAqueous kit and Plant Isolation Aid (Ambion, Austin, TX, USA). For the developmental series looking at the FPIs, tissue was collected at z = 7.5 h in LD and RNA was isolated from plant apices and young leaves using Plant RNA Isolation Reagent (Invitrogen). The RNA was treated with Turbo DNase (Ambion) to remove genomic DNA contamination. cDNA was synthesized from 5 μg of RNA using Superscript II reverse transcriptase (Invitrogen) and oligo (dT) primers. Real time PCR reactions were carried out using the Brilliant II SYBR Green QPCR Master Mix (Stratagene, Cedar Creek, TX, USA/La Jolla, CA, USA) in the Stratagene Mx3005P QPCR System. Each 25 μl reaction included 5 μl of cDNA that had been diluted 1:100 and had a final primer concentration of 300 nM for each primer. A list of primers is included in Additional File 3: Table 4. Standard curves were run for all primer pairs to ensure high efficiency. For *AqGI*, *AqCO*, *AqFT*, and *AqIPP2*, the annealing temperature was 55°C and the extension was 30 seconds. The annealing temperature for *AqAGL24.1*, *AqAGL24.2*, *AqSOC1*, *AqLFY*, and *AqAP1 *was 60°C with a 20-second extension. For each data point, three biological samples were taken for the diurnal expression experiment and six biological samples were taken for each point in the developmental series. Expression for each biological sample was assayed from two replicates per reaction plate with three independent reaction plates run per gene. Efficiency-corrected comparative quantification was used to quantify relative expression using a pool of all sample cDNAs as a calibrator and using *AqIPP2 *(isopentyl pyrophosphate:dimethylallyl pyrophosphate isomerase) expression for normalization. Variability resulting from technical replicates was negligible compared to the variability from biological replicates so only biological variability is presented here.

### *In situ *hybridization

A microwave fixation system was used to fix tissue and prepare for embedding (Microwave Research Applications model BP-111-RS, Laurel, MD, USA). The power setting was 55 for all steps. Collected plant tissue was fixed in ice cold FAA (50 ethanol:35 RNAse free water:10 37% formaldehyde:5 glacial acetic acid; microwave temperature setting 37°C, 3 × 15 minutes on ice). Tissue was then dehydrated through an ethanol series (50%, 70%, 95%, 100% × 2; microwave temperature setting 67°C, 1.2 minutes in room temperature water bath) before clearing in citrosolve (50 ethanol:50 citrosolve,100% citrosolve; microwave temperature setting 67°C, 1.5 minutes in room temperature water bath). Following tissue clearing, tissue was gradually infiltrated with Paraplast Extra (50 citrosolve:50 Paraplast Extra; microwave temperature setting 67°C, 10 minutes in hot water bath, followed by 100% Paraplast Extra × 5, 30 minutes in hot water bath). To aid the infiltration process, tissue was vacuum infiltrated in melted Paraplast Extra for 2 minutes prior to each microwave step. Tissue was then embedded in blocks in Paraplast Extra. Embedded tissue was stored at 4°C until use.

*In situ *probes were designed for each gene (*AqLFY*, *AqTFL*, *AqFL1*, *AqSOC1.1*, *AqAGL24.2*) to be approximately 300 to 400 bp long in the 3′ region of genes, including some of the 3′ UTR. Primers used for probe amplification can be found in Additional File 3: Table 3. Probe fragments were amplified from *Aquilegia formosa *cDNA and cloned into the TOPO TA vector (Invitrogen). Following linearization of the vector, two sets of digoxigenin-labeled probes were transcribed in both the sense and anti-sense orientation using T7 or T3 RNA polymerase (Roche Diagnostics, Indianapolis, IN, USA). One set of probes was alkaline hydrolized to generate smaller probes in the 150 to 200 bp size range while the other set was left full length. A Reichert-Jung microtome and disposable steel blades were used to section samples to 8 μm. RNA *in situ *hybridization was carried out according to the protocol in Kramer *et al*. (2005). Sections were counterstained using 1% calcofluor and imaged using both white and fluorescent light in the Harvard Center for Biological Imaging on a Leica Leitz DMRD microscope (Leica Microsystems, Bannockburn, IL, USA) equipped with a Retiga EXi imaging system (Q Imaging, Surrey, BC, Canada).

## Abbreviations

ACT: Arabidopsis CENTRORADIALIS; AP1: APETALA1; BAC: Bacterial Artificial Chromosome; BFT: BROTHER OF FT; CO: CONSTANS; CRY: CRYPTOCHROME; DN: Day Neutral; EST: Expressed Sequence Tag; FM: Floral Meristem; FMI: Floral Meristem Identity; FPIs: Floral Pathway Integrators; FLC: FLOWERING LOCUS C; FLK: FLOWERING LOCUS K; FT: FLOWERING LOCUS T; GI: GIGANTEA; IPP2: ISOPENTYL PYROPHOSPHATE:DIMETHYLALLYL PYROPHOSPHATE ISOMERASE2; LD: Long Day; LFY: LEAFY; MADS: MCM1/AGAMOUS/DEFICIENS/SRF; MFT: MOTHER OF FT; PEBP: Phosphatidylethanolamine Binding Protein; PHY: PHYTOCHROME; SAM: Shoot Apical Meristem; SD: Short Day; TFL: TERMINAL FLOWER; TSF: TWIN SISTER OF FT; VIGS: Virus Induced Gene Silencing; VIN3: VERNALIZATION INSENSITIVE 3; VRN1: VERNALIZATION 1; VRN2: VERNALIZATION 2.

## Competing interests

The authors declare that they have no competing interests.

## Authors' contributions

ESB and EMK designed the experiments and worked together to draft the manuscript. ESB performed all of the experiments described. Both authors read and approved of the final manuscript.

## Supplementary Material

Additional File 1 **Photoperiod Data**. Figure 1: Basic morphology of plants grown in either LD or SD. **A**. LD- and SD-grown vegetative plants shown from above. LD plants generally have slightly fewer leaves borne on longer petioles and with more expanded laminae than SD plants. **B**. LD- and SD-grown flowering plants shown from the side. LD plants exhibit more internodal expansion in their inflorescences and produce more flowers. Figure 2:Diurnal expression of *AqGI*, *AqCO*, and *AqFT *genes. **A**. Quantitative real-time RT-PCR on *AqGI*, *AqCO *and *AqFT *using tissue samples collected over the span of 24 hours. Each data point is the mean of three biological samples. Error bars are the standard error of the biological samples. In some cases, the error bars are smaller than the point width and cannot be seen. Shaded areas represent darkness. **B**. Summary of diurnal regulation of *GI*, *CO *and *FT *homolog expression in Arabidopsis, *Aquilegia *and *Oryza*. Based on [67,92,93]. While the expression of *GI *orthologs is highly conserved, that of *CO *and *FT *is much less so. One consistency across all three taxa is that expression of *FT *homologs is correlated with promotion of flowering: *FT *is expressed in LD in Arabidopsis, in both LD and SD in day neutral *Aquilegia*, and in SD in *Oryza*. Click here for file

Additional File 2 **Phylogenetic Analyses**. Figure 1: Neighbor joining tree showing the relationships of annotated *Aquilegia *type II MADS-box genes to genes from other taxa (*Arabidopsis, Petunia*, *Oryza *and gymnosperms). Representatives from *Aquilegia *are indicated with a diamond. Subfamilies are identified on the right after Becker and Theissen [42]. Numbers at the major nodes are bootstrap values >50 from 1,000 replicates and asterisks at internal nodes indicate bootstrap support >50. Figure 2: *StMADS11 ***(A) **and *TM3 ***(B) **related MADS-box gene trees. The strict consensus of the most parsimonious trees generated from analysis of amino acid sequence alignments. Numbers at nodes are bootstrap values greater than 50 generated using 1,000 replicates. The tree was rooted using gymnosperm sequences. The two *AqAGL24 *homologs are equally related to Arabidopsis *AGL24 *and Arabidopsis *SVP *with little support for nodes. *AqSOC1 *belongs to the clade including Arabidopsis *SOC1 *with weak support. Figure 3: *FT *gene family tree. The strict consensus of the most parsimonious trees generated from nucleotide sequence alignments. Bootstrap values are presented at nodes when >50. *Aquilegia formosa *and *Arabidopsis thaliana *representatives are in bold. The tree was rooted using *Picea sitchensis *CO226804. Figure 4: *LFY *homolog tree. The strict consensus of the most parsimonious trees generated from amino acid sequence alignments. The tree was rooted with *Psilotum*, *Angiopteris *and *Ceratopteris *sequences. Figure 5: Phytochrome **(A) **and cryptochrome **(B) **homolog trees. The most parsimonious trees generated from amino acid alignments. The phytochrome tree was rooted with the phyA and phyC clades while the cryptochrome tree was rooted such that the cry1 and cry2 clades are each monophyletic. Figure 6: *GIGANTEA *homolog tree. The strict consensus of the most parsimonious trees generated from amino acid sequence alignments. The tree was rooted with the monocot sequences. Figure 7: *CO *gene family tree. The strict consensus of the most parsimonious trees generated from amino acid alignments. Bootstrap values are presented at nodes when >50. *Aquilegia formosa *and *Arabidopsis thaliana *representatives are in bold. The *CO *tree was rooted using *Aquilegia formosa *DR918, *Solanum lycopersicum *BT013685, and *Arabidopsis thaliana COL13 *NM130356.4. Figure 8: *FRIGIDA *and *FRI*-like unrooted tree. Unrooted strict consensus of the most parsimonious trees generated using amino acid alignments. Click here for file

Additional File 3 **Tables**. Table 1: *Arabidopsis *and *Aquilegia *floral development stages. Based on [94] and [95]. Table 2: Gene cloning primer list. Table 3: qRT-PCR primer list. Table 4: qRT-PCR Primer List. Click here for file
