# Supplementary figures and images for: Environmental and molecular analysis of the floral transition in the lower eudicot Aquilegia formosa
Source: EvoDevo. 2011 Feb 17;2:4. doi: 10.1186/2041-9139-2-4 (PMC3049749; doi:10.1186/2041-9139-2-4)

A

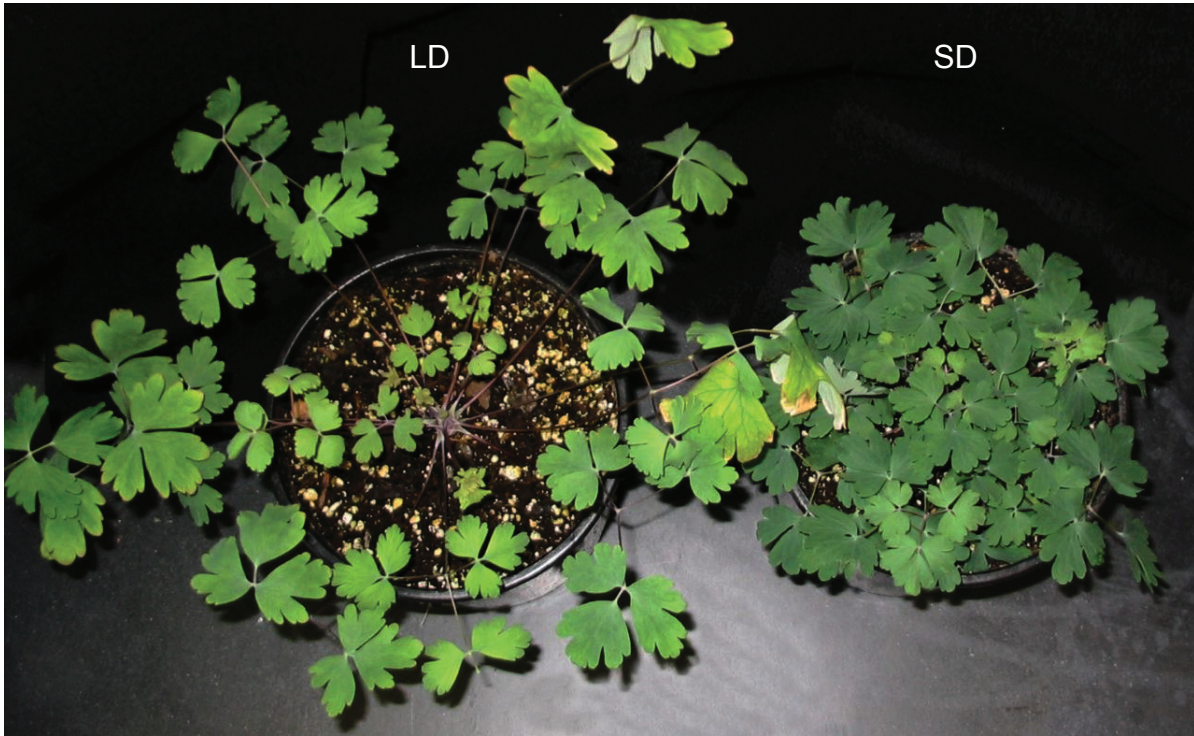

B

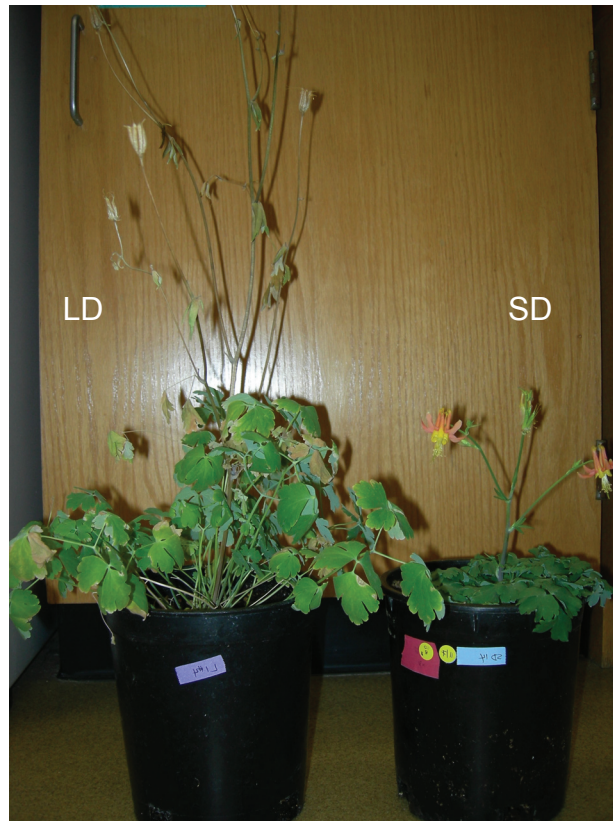

A.

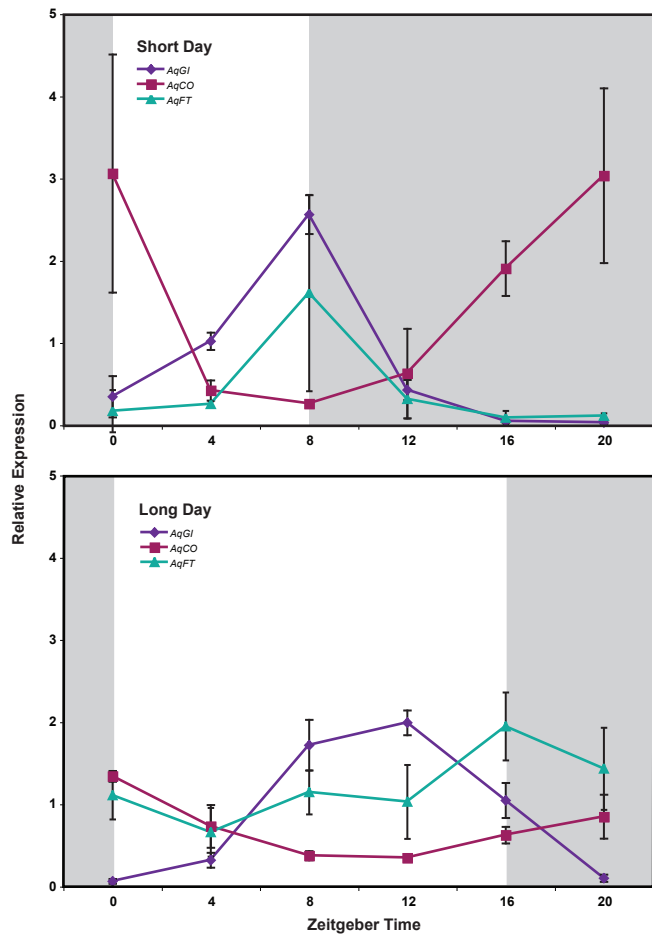

B. Arabidopsis

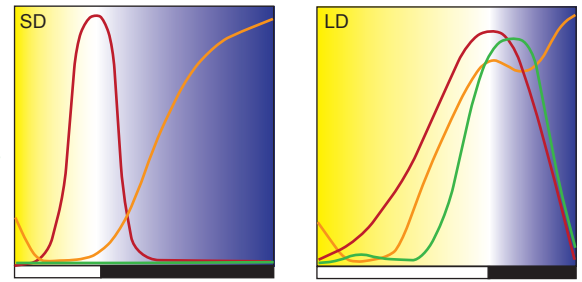

Aquilegia

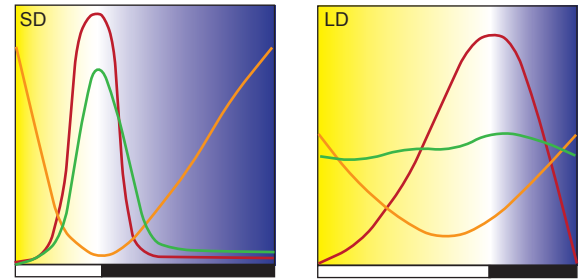

Oryza

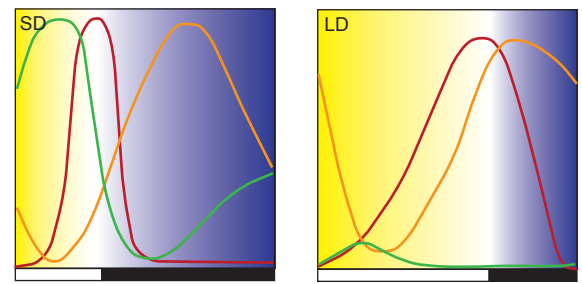

Supplement: Additional File 1 — Photoperiod Data. Figure 1: Basic morphology of plants grown in either LD or SD. A. LD- and SD-grown vegetative plants shown from above. LD plants generally have slightly fewer leaves borne on longer petioles and with more expanded laminae than SD plants. B. LD- and SD-grown flowering plants shown from the side. LD plants exhibit more internodal expansion in their inflorescences and produce more flowers. Figure 2:Diurnal expression of AqGI, AqCO, and AqFT genes. A. Quantitative real-time RT-PCR on AqGI, AqCO and AqFT using tissue samples collected over the span of 24 hours. Each data point is the mean of three biological samples. Error bars are the standard error of the biological samples. In some cases, the error bars are smaller than the point width and cannot be seen. Shaded areas represent darkness. B. Summary of diurnal regulation of GI, CO and FT homolog expression in Arabidopsis, Aquilegia and Oryza. Based on [67,92,93]. While the expression of GI orthologs is highly conserved, that of CO and FT is much less so. One consistency across all three taxa is that expression of FT homologs is correlated with promotion of flowering: FT is expressed in LD in Arabidopsis, in both LD and SD in day neutral Aquilegia, and in SD in Oryza. [file 2041-9139-2-4-S1.PDF]
