# Supplementary material for: Environmental and molecular analysis of the floral transition in the lower eudicot Aquilegia formosa
Source: EvoDevo. 2011 Feb 17;2:4. doi: 10.1186/2041-9139-2-4 (PMC3049749; doi:10.1186/2041-9139-2-4)
Supplement: Additional File 2 — Phylogenetic Analyses. Figure 1: Neighbor joining tree showing the relationships of annotated Aquilegia type II MADS-box genes to genes from other taxa (Arabidopsis, Petunia, Oryza and gymnosperms). Representatives from Aquilegia are indicated with a diamond. Subfamilies are identified on the right after Becker and Theissen [42]. Numbers at the major nodes are bootstrap values >50 from 1,000 replicates and asterisks at internal nodes indicate bootstrap support >50. Figure 2: StMADS11 (A) and TM3 (B) related MADS-box gene trees. The strict consensus of the most parsimonious trees generated from analysis of amino acid sequence alignments. Numbers at nodes are bootstrap values greater than 50 generated using 1,000 replicates. The tree was rooted using gymnosperm sequences. The two AqAGL24 homologs are equally related to Arabidopsis AGL24 and Arabidopsis SVP with little support for nodes. AqSOC1 belongs to the clade including Arabidopsis SOC1 with weak support. Figure 3: FT gene family tree. The strict consensus of the most parsimonious trees generated from nucleotide sequence alignments. Bootstrap values are presented at nodes when >50. Aquilegia formosa and Arabidopsis thaliana representatives are in bold. The tree was rooted using Picea sitchensis CO226804. Figure 4: LFY homolog tree. The strict consensus of the most parsimonious trees generated from amino acid sequence alignments. The tree was rooted with Psilotum, Angiopteris and Ceratopteris sequences. Figure 5: Phytochrome (A) and cryptochrome (B) homolog trees. The most parsimonious trees generated from amino acid alignments. The phytochrome tree was rooted with the phyA and phyC clades while the cryptochrome tree was rooted such that the cry1 and cry2 clades are each monophyletic. Figure 6: GIGANTEA homolog tree. The strict consensus of the most parsimonious trees generated from amino acid sequence alignments. The tree was rooted with the monocot sequences. Figure 7: CO gene family tree. The strict consensus of [file 2041-9139-2-4-S2.PDF]

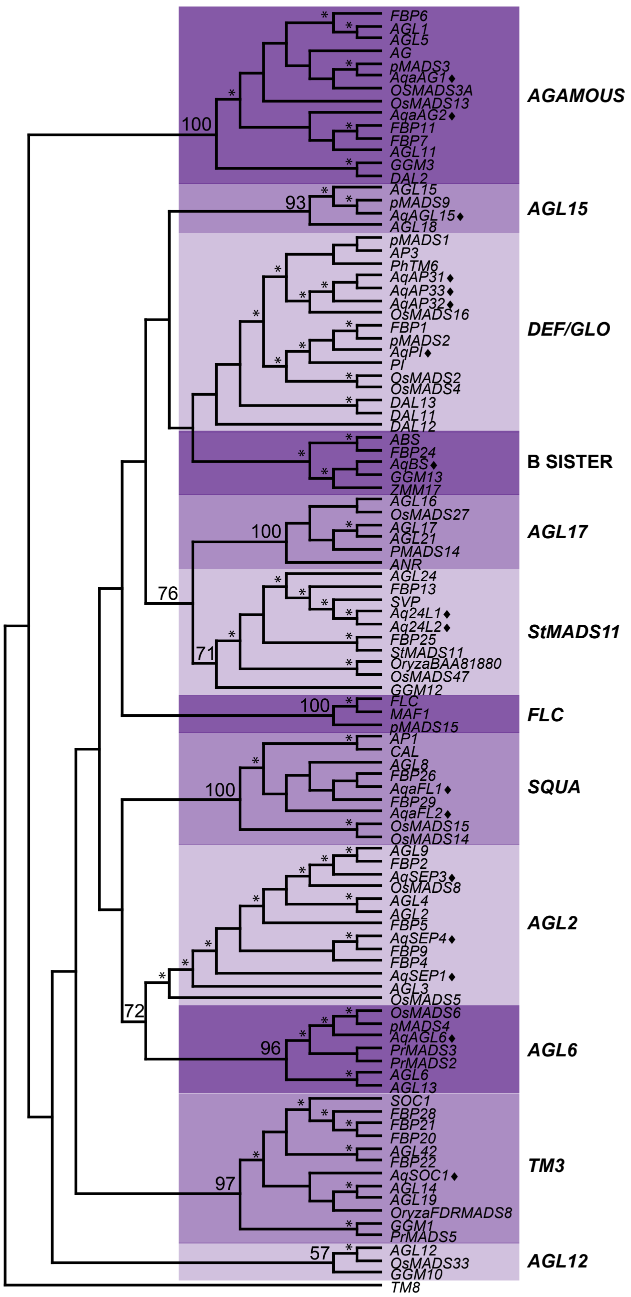

Additional File 2 - Figure 1

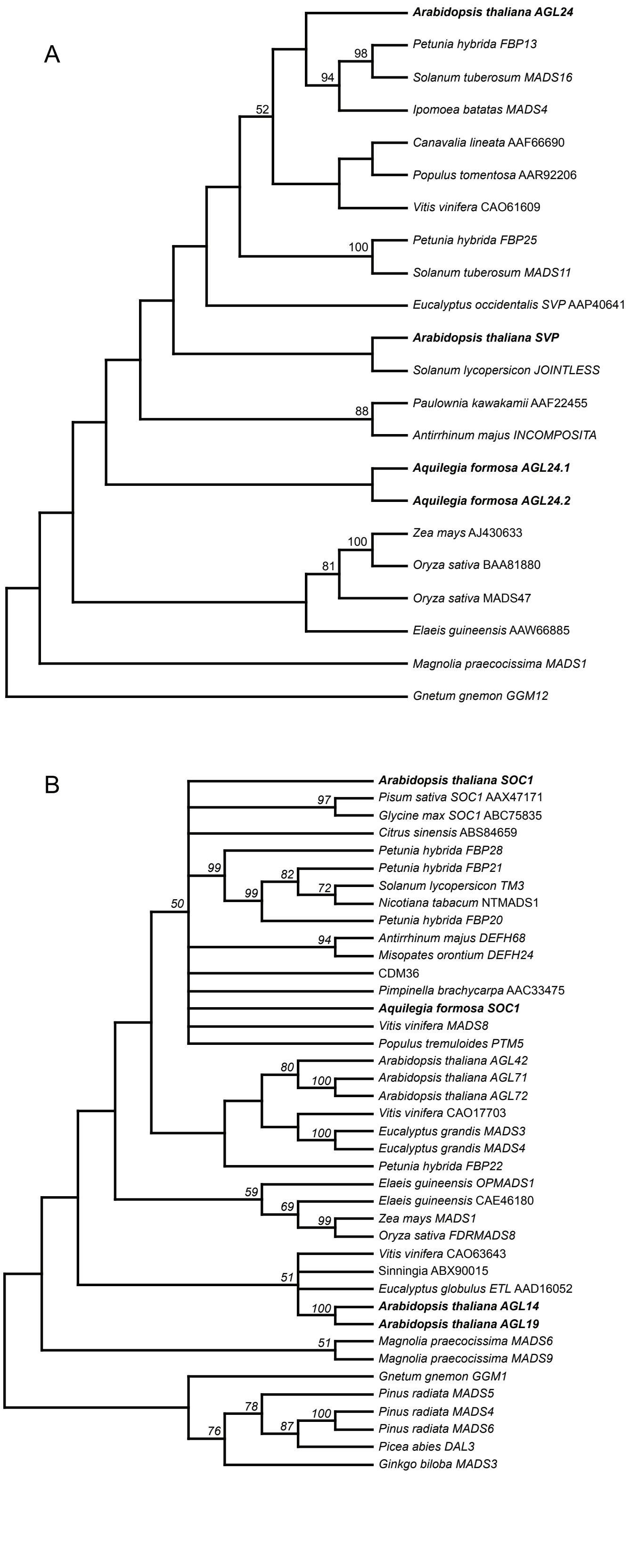

Additional File 2 - Figure 2

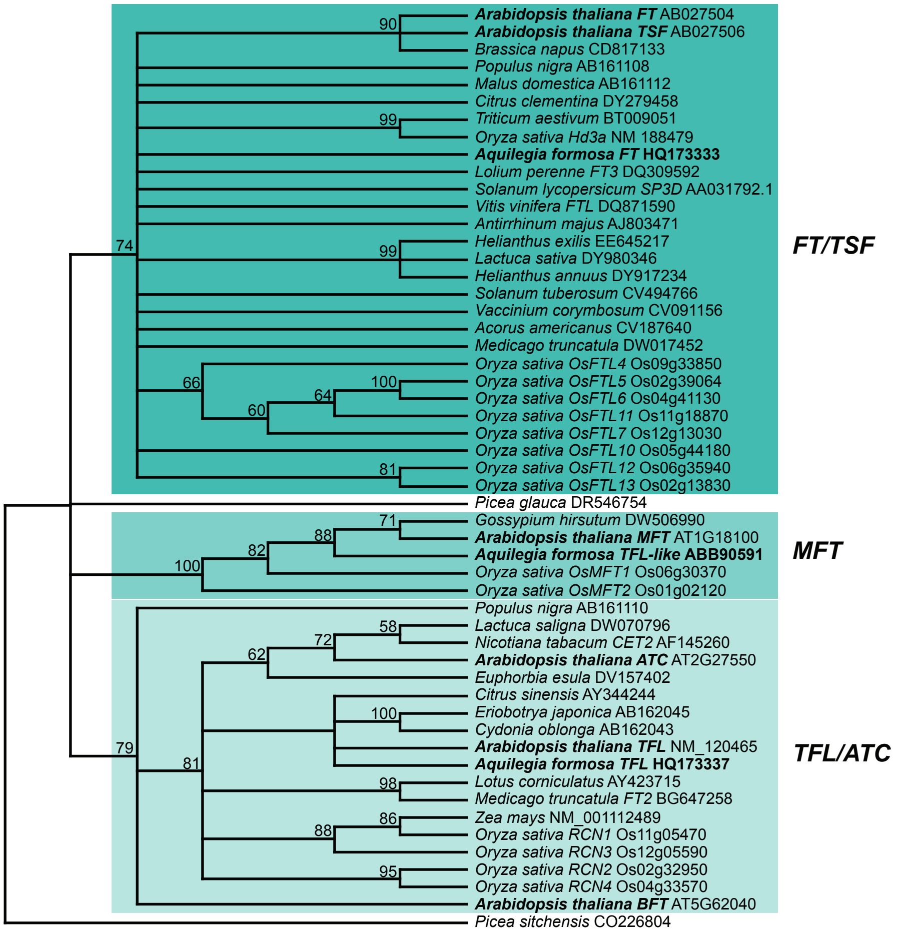

Additional File 2 - Figure 3

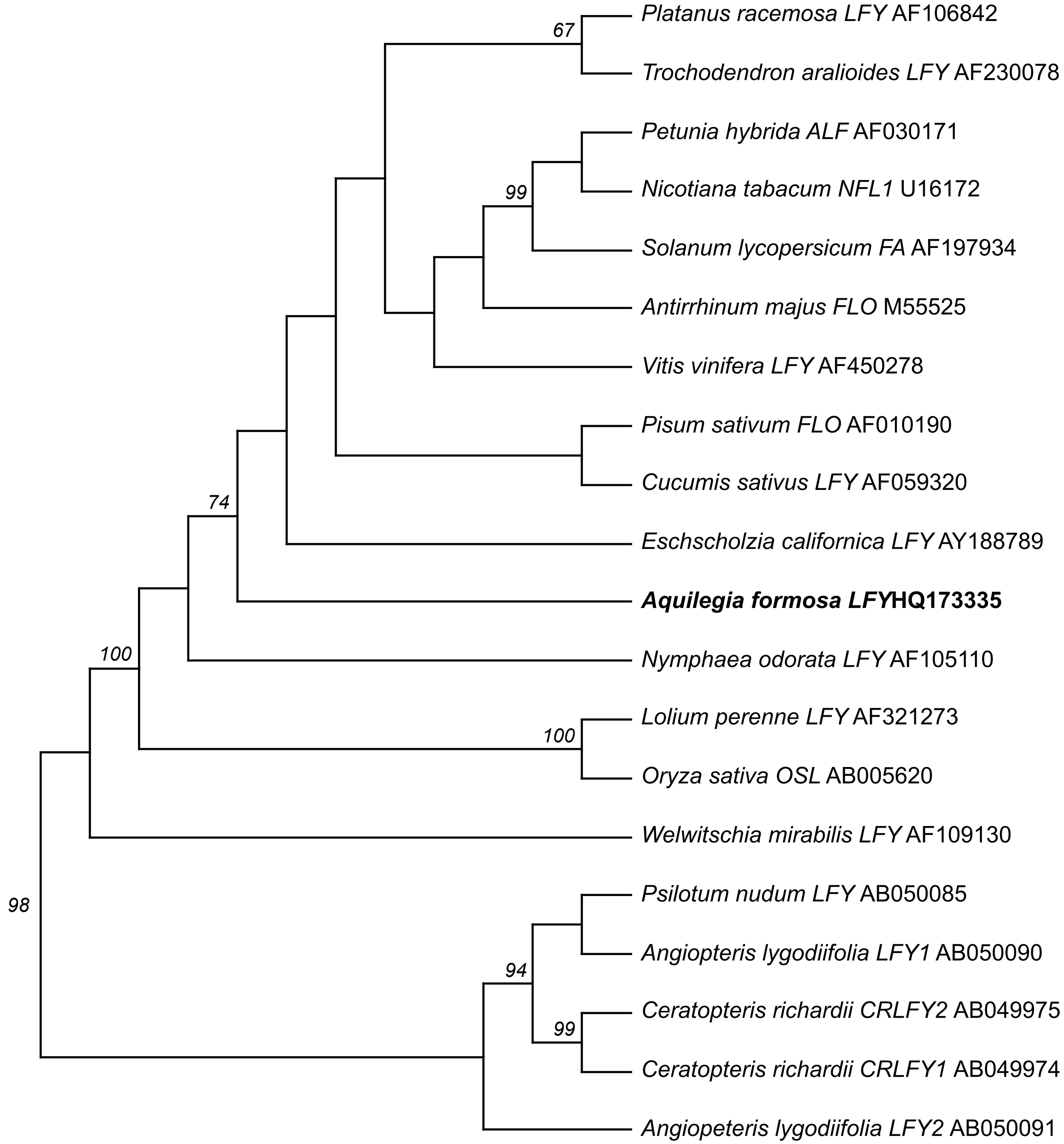

Additional File 2 - Figure 4

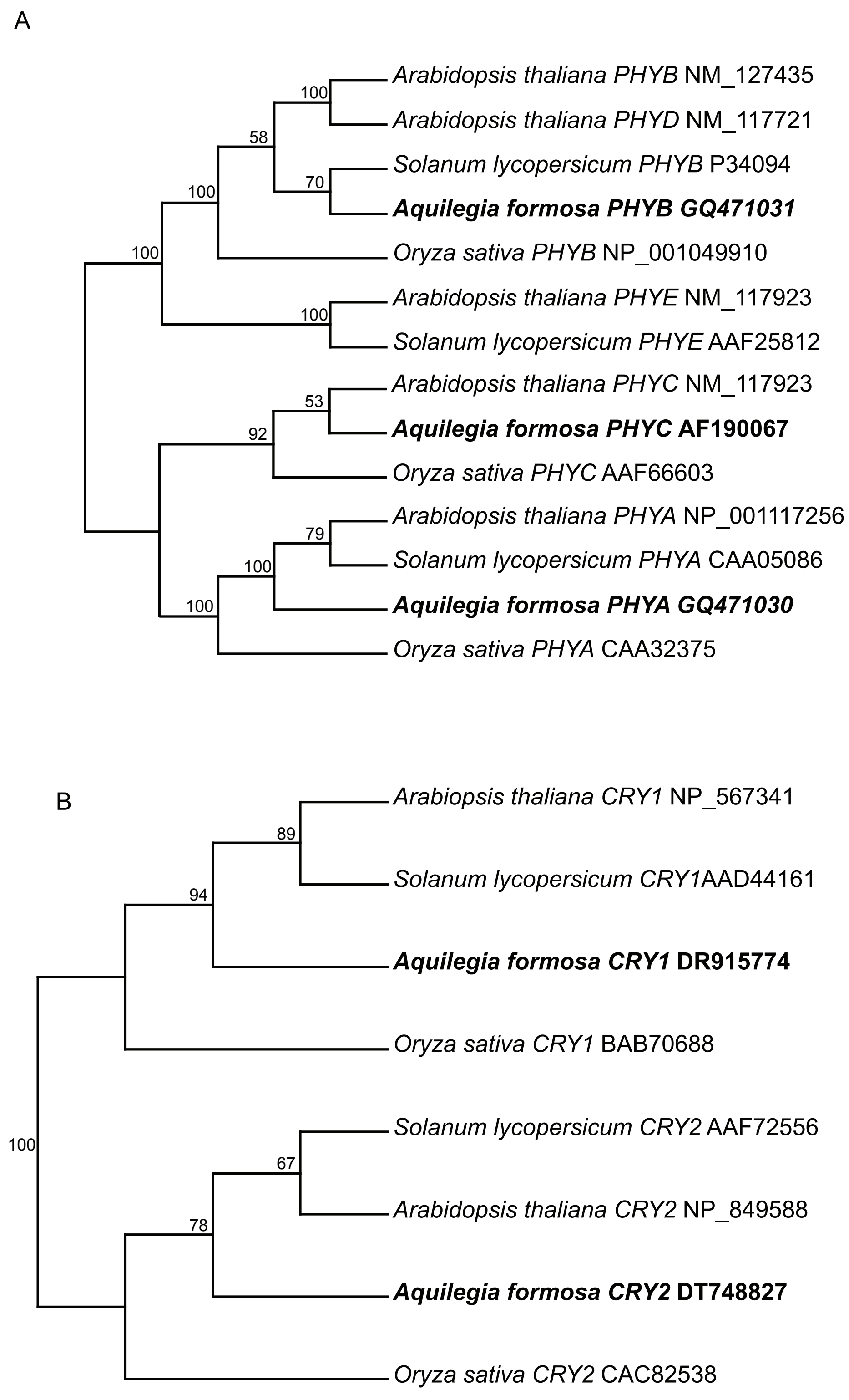

Additional File 2 - Figure 5

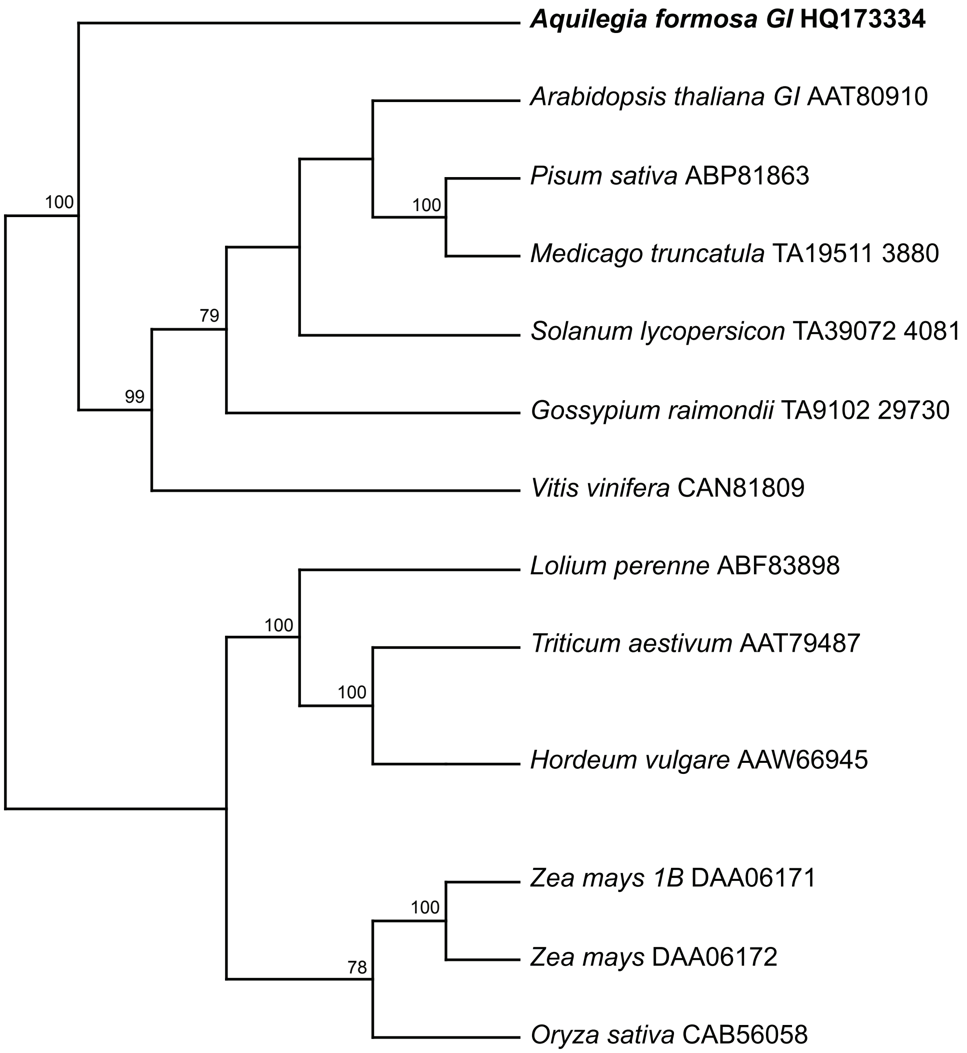

Additional File 2 - Figure 6

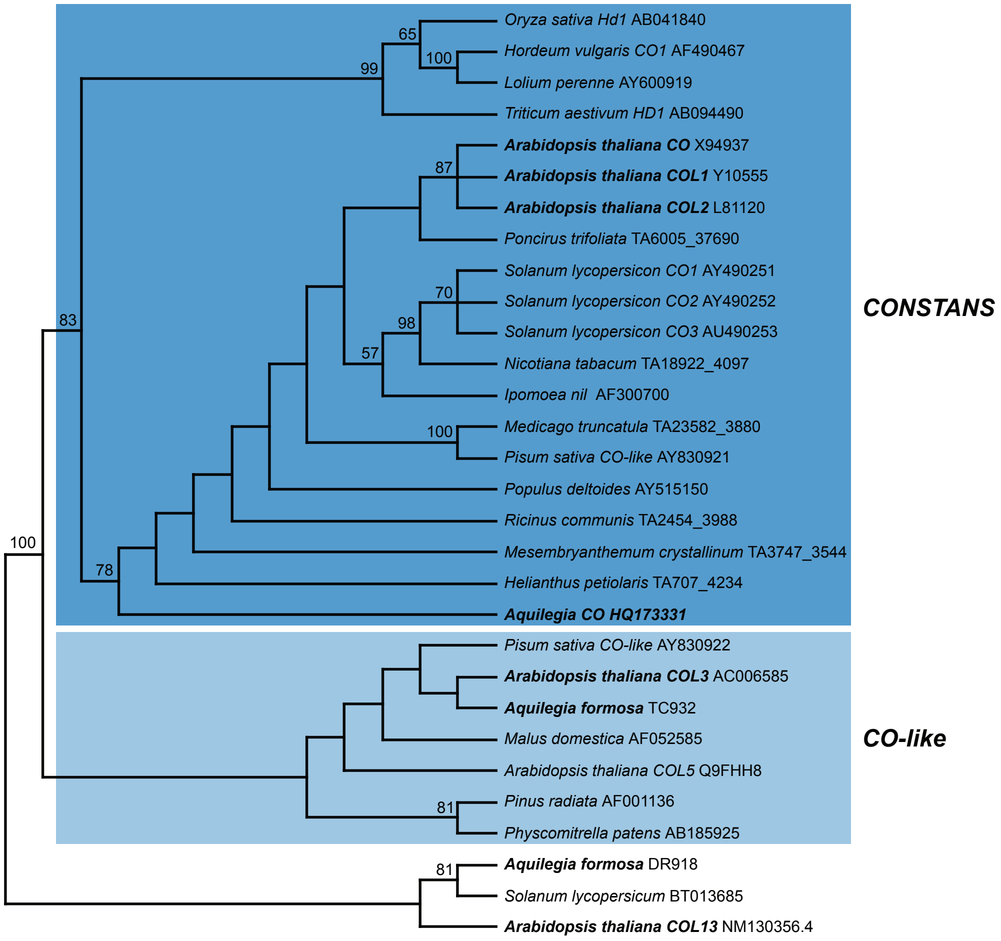

Additional File 2 - Figure 7

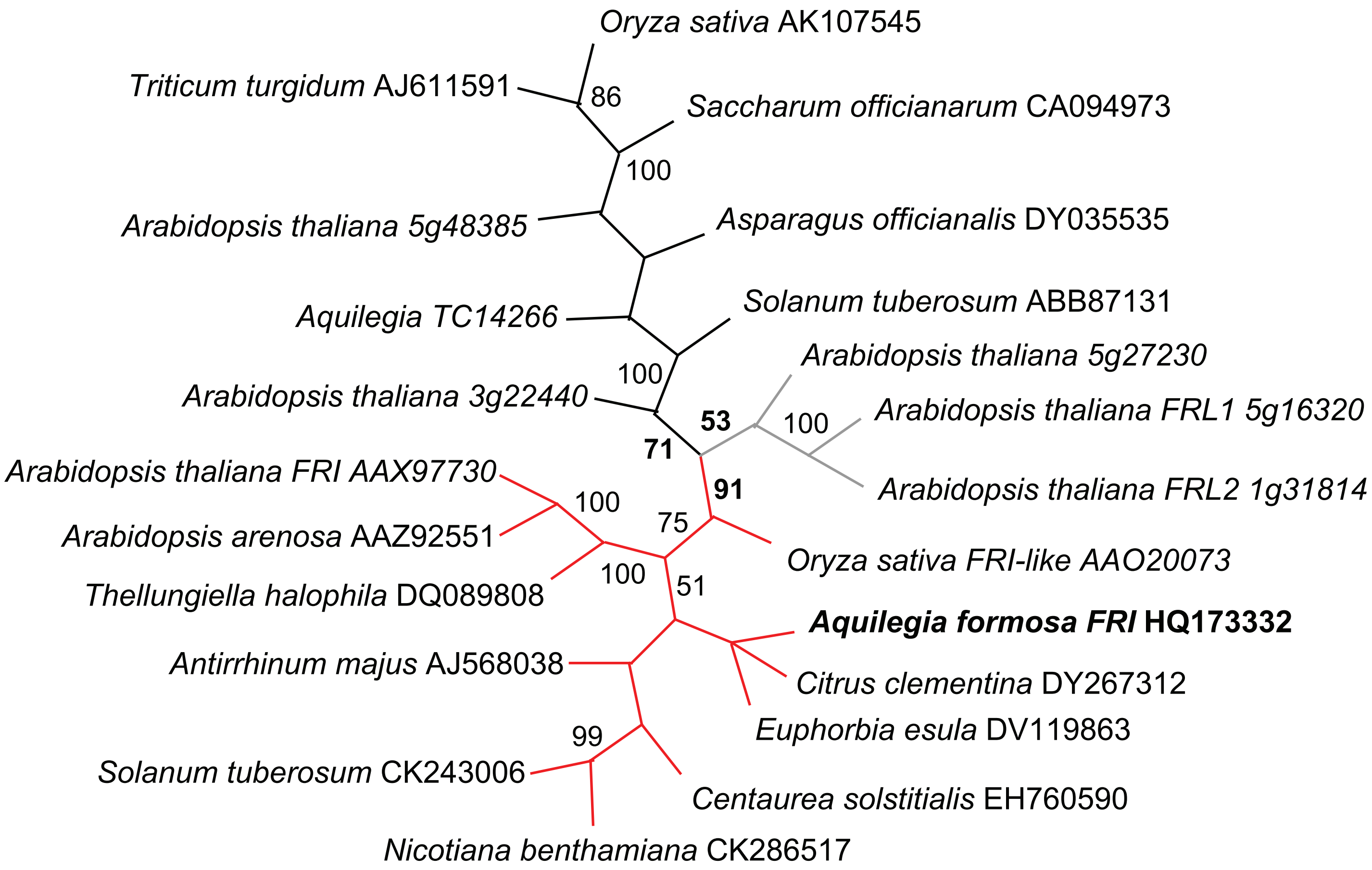

Additional File 2 - Figure 8
