# Supplementary material for: Environmental and molecular analysis of the floral transition in the lower eudicot Aquilegia formosa
Source: EvoDevo. 2011 Feb 17;2:4. doi: 10.1186/2041-9139-2-4 (PMC3049749; doi:10.1186/2041-9139-2-4)
Supplement: Additional File 3 — Tables. Table 1: Arabidopsis and Aquilegia floral development stages. Based on [94] and [95]. Table 2: Gene cloning primer list. Table 3: qRT-PCR primer list. Table 4: qRT-PCR Primer List. [file 2041-9139-2-4-S3.PDF]

Table 2 - Arabidopsis and *Aquilegia* Floral Development Stages		
**Stage**	**Arabidopsis**[1]	***Aquilegia***[2]
1	Floral buttress arises	Pre-floral meristem arises (Fig. 2A)
2	Flower primordium forms	Two bracteole meristem (Fig. 2C)
3	Sepal primordia arise	Sepals arise (true floral meristem) (Fig. 2D-F)
4	Sepals overlie flower meristem	Petal primordia arise (Fig. 2G-H)
5	Petal and stamen primordia arise	Stamen primordia begin arising (Fig. 2I-K)
6	Sepals enclose bud	Sepals enclose bud, stamen primordia continue appearing (Fig. 2L-M)
7	Long stamen primordia stalked at base	Carpels initiate, staminodia distinguishable, first whorl of stamens becoming stalked at base (Fig. 2N-P)
8	Locules appear in long stamens	Petal primordia begin to differentiate, first whorl of stamens begin to differentiate locules, folded carpels remain open (Fig. 3A-D)
9	Petal primordia stalked at base	Petals continue to differentiate and elongate to same length as first whorl stamens, all stamens differentiating, staminodia still filamentous, carpels elongated to same height as innermost stamens but remain open, staminodia begin to flatten (Fig. 3E-H)
10	Petals level with short stamens	Spur formation initiates on petals, all organs elongating (Fig. 3I, K)
11	Stigmatic papillae appear	Spur elongation continues, carpels close, stamens become apiculate (Fig. 3M)
12	Petals level with long stamens	Spurs and all floral organs reach final length
13	Bud opens, petals visible, anthesis	Sepals undergo final expansion and reflex, anthesis

1. Arabidopsis staging from Smyth et al. 1990.
2. *Aquilegia* figure references to Tucker and Hodges 2005.

Table 3 - Gene Cloning Primer List	
**Primer Name**	**5´-3´ Sequence**
AqAGIntSeq2.1	AAGAAGGCTTGCACAGAC
AqAGIntSeq2.2	GTCTGTGCAAGCCTTCTT
AqAGIntSeq1.1	TGGTTGTGGTCTCTCAATAC
AqAGIntSeq1.2	GTATTGAGAGACCACAACCA
AqFTfor	TTYTAYACIRTIGTIATGGTIGAYCCIGA
AqFTrev	CCAICCIGGIGCRTAIACIGTYTGICKICC
AqFT5'rev	CCCTGGAATATCCGTCACCAACCAATGC
AqFT5'rev2	ACAGGCAGACCCAGGTTGTAAAGC
AqFT3'for	CTACTTTTGGACAAGAGGTGG
COFOR	AAYCGIGTIGCITCICGICAYGARCGIGT
COREV	TTYTCYYTRTAYCTIAGIACYCTIGCYTCYCT
CO3'for2	GCCTGTTCAGTATGGGAGTATGG
AqCOfor3'	ACAGCAGCAACAACAGCAGAACA
AqCO5'rev	GGCCTACTCGGGTAATTAGCCGAAGGTCCAT
LFsx1-3	GCCGGCIMGIGGIAARAAYGGIYTIGA
LFtxr	CCTGCCIACRTARTGICKCATYTTIGGYTT
AQALFYrev5'	GAGTTGCACCAGCCTGAATTGCATGTCGATA
AQLFYfor	TTACTTGTTTCATCTCTATGAAGAATGTAG
AqSOC1FOR	ACIAGYAGRCARGTIACITTYTCIAA
AqSOC1revcor	CCIATRAAIARITWIGTYTCIACITC
SOC1for2	ACIAGYMGICARGTIACITTYTCIAA
AqSOC1for3'	GTTGCTGTTATTGTTTTCTCACCA
AqSOC1for3'2	TGACAAGTGTGGAGTGCGAC
AqAGL24FOR	GCIMGRCARGTIACITTYTCIAA
AqAGL24REV	CCIARYTTIARICAIGTRTCIGA
AGL24for2	MGRMGRGGIMTITTYAARAARGCIGA
AqAGL24-2for3'-2	CGAGGAAGGGCAGTCATCAGAGTC
AqAGL24-2for3'	GATATGTCCAGTGAACAAGGAAATG
FRI seq1 for	CGACGCTCATCTTCATTCTCC
FRI seq1 rev	GTTGTCAATGGTAGAGGAACATGC
Aq TFL for	CCIAGYGAYCCITAYCTIARRGARCA
Aq TFL rev	ARCGICTICTIGCIGCRGTYTC
TFL 5' rev 1	GGCATCTCATAGCTTACCACTTCCCTCCC
TFL 5' rev 2	CCCAAATGTAGCATCAGTTGTGCCTGG
TFL 3' for 1	CCAGGCACAACTGATGCTACATTTGGG
TFL 3' for 2	GGGAGGGAAGTGGTAAGCTATGAGATGCC

Table 4 - qRT-PCR Primer List	
**Primer Name**	**5´-3´ Sequence**
AqGI QPCR for	TTCACCAGCAAAGCATCAGC
AqGI QPCR rev	TCAACAAGCATTCCATCCGTG
AqCO QPCR for	TCAGCATCACCACCAGCATAATC
AqCO QPCR rev	TGGCATTTGAAGGGGAGGAC
AqFT QPCR for	TCAAACCCTCTGTTGTTGTTAGCC
AqFT QPCR rev	CGTAGCAAACCACCTCTTGTCC
AqLFYqRTfor	ACACCACTAACCCTCTTGACGC
AqLFYqRTrev	TTTTCATCTCCGCTATTCCTCG
AqFUL1qRTfor	AGGAAAAGGAGAAGGAACTGGC
AqFUL1qRTrev	CGGGTTCCTTCCTCTTCAGC
AqAGL241qRTfor	ATGGCTATTGACTCAGATGTTGTTG
AqAGL241qRTrev	GCTTCCTTCTCACTACATTTTCAGC
AqAGL242qRTfor	GAATGCTACTGATGGAAGAGAATGAA
AqAGL242qRTrev	AGTGATAACCCCAACCTAAGAGAGG
AqSOC1qRTfor	GAAAAGCCTAAGCATCATCAGGG
AqSOC1qRTrev	GATGTTCTGGTTTCATTCTCCACC
AqIPP2 for	CAGGTGAAGACGGACTGAAGTTAT
AqIPP2 rev	CCAAGACTGGAAAAAAGACCACAC
